# RNA Polymerase III Subunit Mutations in Genetic Diseases

**DOI:** 10.3389/fmolb.2021.696438

**Published:** 2021-07-30

**Authors:** Elisabeth Lata, Karine Choquet, Francis Sagliocco, Bernard Brais, Geneviève Bernard, Martin Teichmann

**Affiliations:** ^1^ Bordeaux University, Inserm U 1212, CNRS UMR 5320, ARNA laboratory, Bordeaux, France; ^2^ Department of Genetics, Harvard Medical School, Boston, MA, United States; ^3^ Montreal Neurological Institute, McGill University, Montreal, QC, Canada; ^4^ Departments of Neurology and Neurosurgery, Pediatrics and Human Genetics, McGill University, Montreal, QC, Canada; ^5^ Department of Specialized Medicine, Division of Medical Genetics, McGill University Health Center, Montreal, QC, Canada; ^6^ Child Health and Human Development Program, Research Institute of the McGill University Health Center, Montreal, QC, Canada

**Keywords:** RNA polymerase III (Pol III), Pol III-related hypomyelinating leukodystrophy (POLR3-HLD), innate immunity, neurodegenerative disease, Pol III subunits (POLR3A, POLR3B, POLR3C, POLR3E, POLR3F, POLR3GL, POLR3H, POLR3K, POLR1C)

## Abstract

RNA polymerase (Pol) III transcribes small untranslated RNAs such as 5S ribosomal RNA, transfer RNAs, and U6 small nuclear RNA. Because of the functions of these RNAs, Pol III transcription is best known for its essential contribution to RNA maturation and translation. Surprisingly, it was discovered in the last decade that various inherited mutations in genes encoding nine distinct subunits of Pol III cause tissue-specific diseases rather than a general failure of all vital functions. Mutations in the POLR3A, POLR3C, POLR3E and POLR3F subunits are associated with susceptibility to varicella zoster virus-induced encephalitis and pneumonitis. In addition, an ever-increasing number of distinct mutations in the POLR3A, POLR3B, POLR1C and POLR3K subunits cause a spectrum of neurodegenerative diseases, which includes most notably hypomyelinating leukodystrophy. Furthermore, other rare diseases are also associated with mutations in genes encoding subunits of Pol III (POLR3H, POLR3GL) and the BRF1 component of the TFIIIB transcription initiation factor. Although the causal relationship between these mutations and disease development is widely accepted, the exact molecular mechanisms underlying disease pathogenesis remain enigmatic. Here, we review the current knowledge on the functional impact of specific mutations, possible Pol III-related disease-causing mechanisms, and animal models that may help to better understand the links between Pol III mutations and disease.

## Introduction

Transcription is essential to make genome-encoded information accessible, which is a basic condition for the creation of all life forms. It represents the first step in gene expression and is coordinated by regulatory mechanisms allowing cells to respond not only according to their own needs, but also, if necessary, to demands from neighboring cells or to differentiation programs.

**FIGURE 1:**
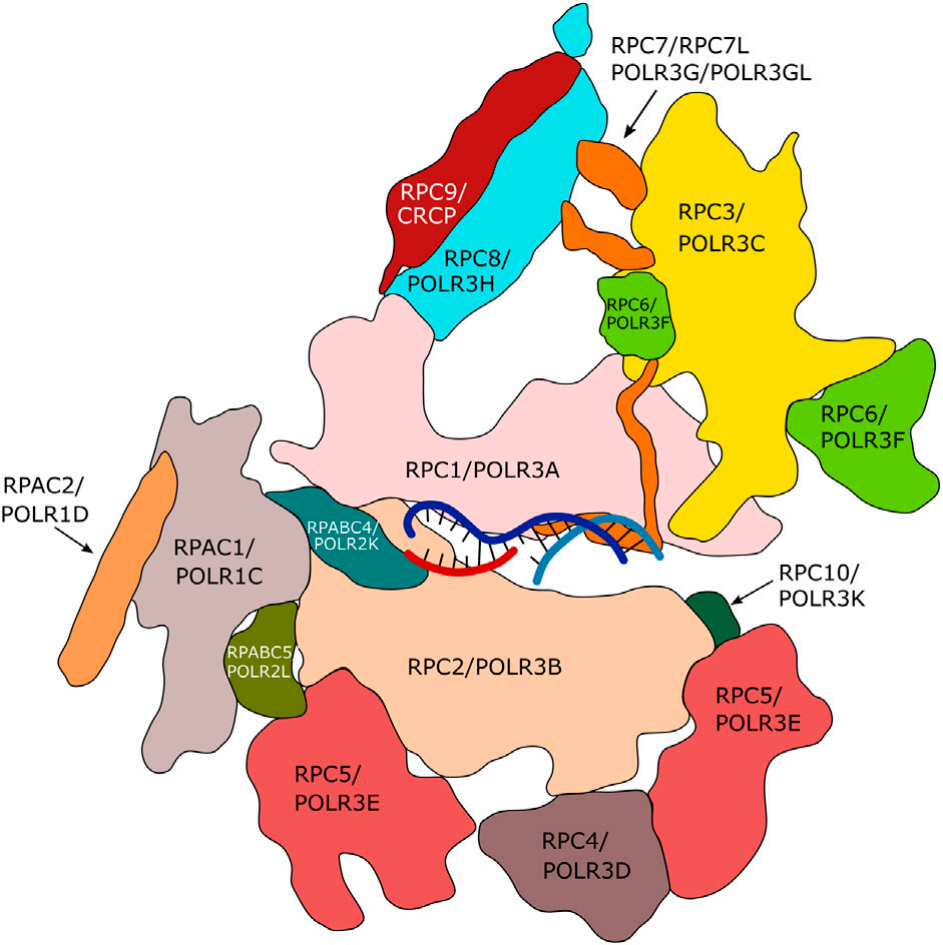
Subunits of human RNA polymerase III. Fourteen of the seventeen subunits of human Pol III are appropriately assigned. Subunits POLR2E, POLR2F and POLR2H are not displayed. The Figure is inspired by the structure of human Pol III published by Girbig et al. (2021) (PDB7AE3). The RPC4/RPC5 hetero-dimeric complex is required by termination/reinitiation. The RPC3/RPC6/RPC7 hetero-trimeric subcomplex is required for transcription initiation.

Nuclear RNA polymerases are responsible for the transcription of genomic DNA into RNA. In eukaryotes, up to five different nuclear DNA-dependent RNA polymerases (Pol I-V) have been described, each of which transcribes specific groups of genes. RNA polymerases I to III are expressed by all eukaryotes. RNA polymerase (Pol) I transcribes the large ribosomal gene, which is present in up to several hundred copies within eukaryotic genomes. The resulting ribosomal (r)RNAs represent the major constituents of ribosomes (Khatter et al., 2017). Pol II is responsible for transcription of all protein-coding genes and is also involved in the expression of several non-coding RNAs (Roeder, 2019 and references therein). Pol III synthesizes a variety of small (<350 nt) and highly expressed RNAs (e.g. 5S rRNA, transfer RNA (tRNA), U6 RNA) that do not code for proteins (Dieci et al., 2007). RNA polymerases IV and V, which have been described exclusively in plants, are involved in RNA-dependent gene silencing (Zhou and Law, 2015). In terms of protein composition, Pol III is the most complex enzyme performing DNA-dependent transcription in eukaryotic cells. It is composed of 17 subunits (in contrast to 14 subunits in Pol I and 12 subunits in Pol II; Figure 1).

Here, we will review recent discoveries connecting Pol III (also referred to as POLR3) transcription to diseases. We will focus on mutations in genes encoding subunits of the Pol III transcription system that have been associated with microbial infections or with neurodegenerative diseases, including Pol III-related hypomyelinating leukodystrophy (POLR3-HLD). These mutations are also referred to as pathogenic variants in medical genetics. First, we will give an overview of the regulation of Pol III expression. Subsequently, we will review mutations in genes encoding Pol III subunits that were shown to be altered in disease and discuss potential underlying pathophysiological mechanisms that may depend on altered expression of Pol III transcripts. Finally, we will discuss the role of Pol III in innate immunity and related diseases.

## Transcription by RNA Polymerase III

### The Pol III Promoter Types

**FIGURE 2:**
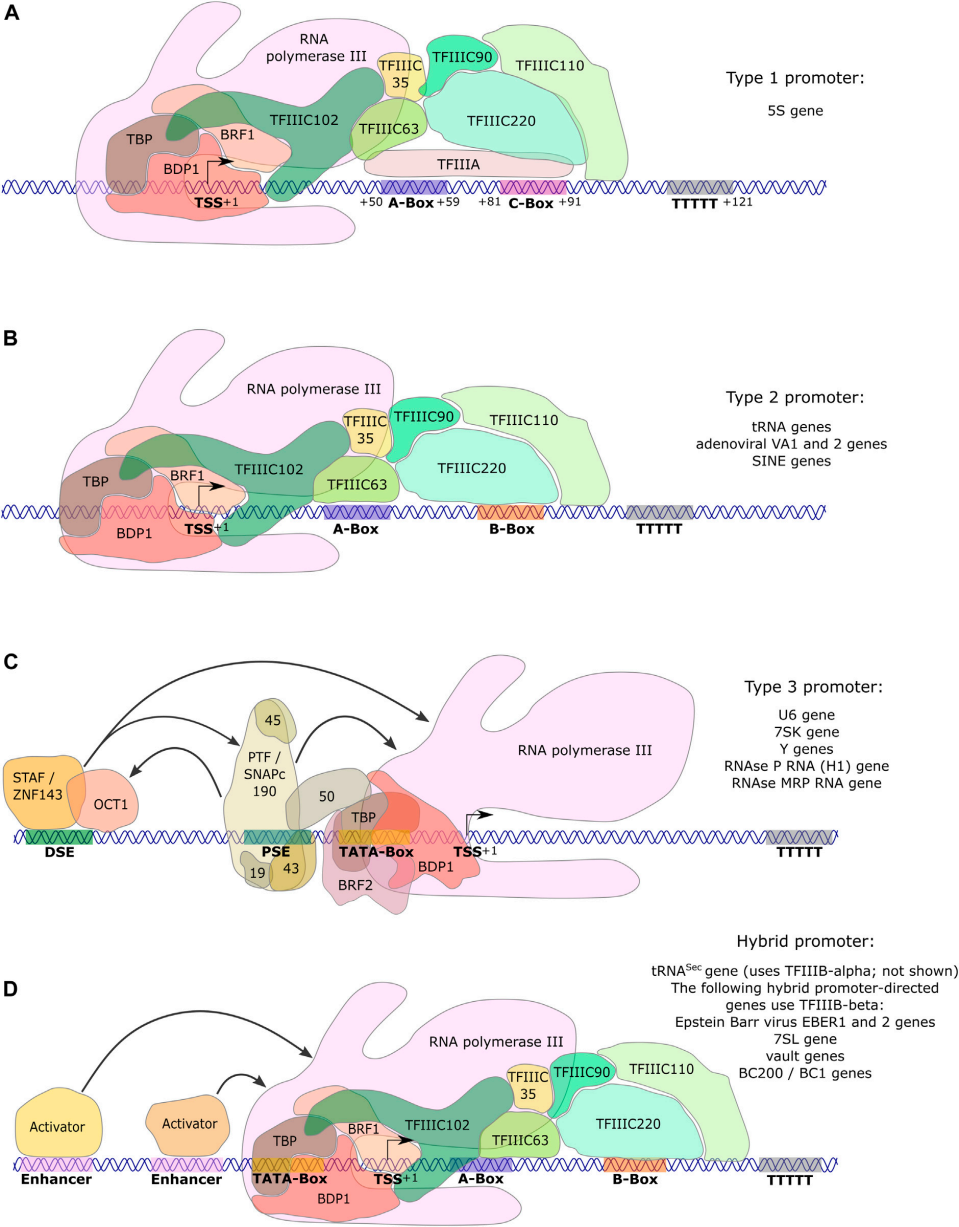
Promoters directing human RNA polymerase III transcription. Pol III type 1 and type 2 genes contain gene-internal promoter elements. **(A)** Type 1 gene transcription of 5S ribosomal (r)RNA is directed by A- and C-boxes that are located relative to the transcription start site (TSS) as indicated. These promoter elements are bound by TFIIIA, permitting the recruitment of TFIIIC and subsequently of TFIIIB-β (composed of TBP, BDP1 and BRF1), which altogether recruit Pol III. **(B)** Type 2 genes (tRNA genes, VA1, VA2, SINEs) contain A- and B-boxes as promoter elements at varying positions relative to the TSS. They are bound by TFIIIC, subsequently allowing recruitment of TFIIIB-β and in turn of Pol III. **(C)** Type 3 gene regulatory elements are entirely located upstream (5′) of the TSS. They are composed of a TATA-box at -30, as well as a proximal sequence element (PSE) and a distal sequence element (DSE) at variable distances with respect to the TSS, depending on the gene. Transcriptional activators (STAF/ZNF143; SNAPc/PTF) bind to the DSE and PSE, respectively, and regulate transcriptional activity. The TATA-box is the only promoter element required for directing Pol III to the TSS (Teichmann et al., 1997). It is bound by TFIIIB-α (composed of TBP, BDP1 and BRF2), which in turn recruits Pol III. **(D)** Hybrid promoter-directed transcription is regulated by gene-internal elements of type 2 promoters (A- and B-boxes) and additionally by gene regulatory elements upstream of the TSS. All these elements vary in their distance to the TSS depending on the gene. In the presence of the PSE (tRNA^Sec^ gene), TFIIIB-α is recruited (not shown in the Figure). In the case of all other enhancer-activator combinations with gene-internal A- and B-boxes (EBER 1 and 2; 7SL; vault; BC1 and BC200), TFIIIB-β is recruited, allowing the subsequent recruitment of RNA polymerase III. For all promoter types, the stretch of T’s represents the transcription termination site. Arrows in panels C. and D. symbolize protein-protein-interactions that contribute to activation of Pol III transcription from these promoters. Promoter types were reviewed in Dumay-Odelot et al. (2014).

Three main promoter types are employed by Pol III: types 1 and 2 have gene internal elements, while type 3 possesses regulatory elements in the 5′ region upstream of the transcriptional start site (TSS) (Figures 2A–C; reviewed in Dumay-Odelot et al. (2014)). The type 1 promoter, consisting of an A- and C-Box, is exclusively used by the 5S rRNA genes (Figure 2A). Expression of tRNA and the adenoviral VA1 and VA2 genes depends on type 2 promoters, which are comprised of A- and B-Boxes. Furthermore, type 2 promoters are encountered in short interspersed nuclear elements (SINEs) (Figure 2B). Type 3 promoters regulate transcription of the U6 small nuclear (sn) RNA, the H1 RNA component of RNase P, the RNA component of RNase MRP, Y RNAs and the 7SK RNA (Dieci et al., 2007; Figure 2C). The type 3 gene regulatory elements include a TATA-box, a proximal sequence element (PSE) and a distal sequence element (DSE), which are respectively located approximately 30, 50 and 200 nt upstream of the TSS. This promoter type emerged during evolution from single cell to multicellular eukaryotes and has been accompanied by the appearance of new transcription factors (Teichmann et al., 2010; Girbig et al., 2021). In addition, there are promoter variations, which are composed of combinations of regulatory elements from type 2 and 3 promoters, as well as of enhancer elements that are known from Pol II transcription. Such hybrid promoter-dependent genes include the selenocysteine tRNA gene (tRNA^Sec^), the Epstein Barr virus EBER gene, the 7SL RNA gene, vault RNA genes (Howe and Shu, 1989; Bredow et al., 1990a,b; Carbon and Krol, 1991; Kickhoefer et al., 2003) and the BC200 RNA gene (Khanam et al., 2007) (Figure 2D).

### The Pol III Transcription Factors

Expression of genes regulated by intragenic promoters requires the six subunit transcription factor TFIIIC (type 1 and 2 promoters) and the transcription factor TFIIIA (type 1 promoter only) to recruit the transcription initiation factor TFIIIB-β (Figures 2A,B). The regulatory elements upstream of the TSS in type 3 and the promoter of the selenocysteine tRNA (tRNA^Sec^) gene are recognized by STAF/ZNF143 and OCT1 (DSE), as well as by SNAPc/PTF (PSE), which stimulate the recruitment of TFIIIB-α to the TSS, whereupon Pol III is recruited (reviewed in Schramm and Hernandez (2002), Dumay-Odelot et al. (2010); Figure 2C). TFIIIB-α is composed of the TATA-binding protein (TBP), the B double prime 1 (BDP1) component and the TFIIB-related factor 2 (BRF2), whereas TFIIIB-β contains the TFIIB-related factor 1 (BRF1) instead of BRF2 (Figure 2) (Teichmann and Seifart, 1995; Teichmann et al., 2000; Schramm et al., 2000; reviewed in Schramm and Hernandez (2002), Dumay-Odelot et al. (2010)). Hybrid promoters display gene-specific transcription factor requirements. While transcription of the 7SL and EBER genes is stimulated by binding of the Pol II transcriptional activator ATF upstream of the TATA-like box, activation of the tRNA^Sec^ gene is dependent on transcription factors that recognize the PSE and DSE (SNAPc/PTF, STAF/ZNF143; Schaub et al., 2000; Figure 2D), which also regulate the transcription rate of type 3 promoters in multicellular organisms (Carbon and Krol, 1991; Meissner et al., 1994; reviewed in Dieci et al. (2007), Dumay-Odelot et al. (2010)). Furthermore, only the promoters that depend on a PSE and SNAPc/PTF transcription factors recruit the BRF2-containing TFIIIB-α transcription initiation factor, whereas other enhancer-activator combinations with gene-internal A- and B-Boxes result in the recruitment of the BRF1-containing TFIIIB-β.

### Pol III Transcription: High Efficiency Through Compact Gene Organization

Human genes transcribed by Pol III are composed of maximally ∼300–350 nucleotides, with the longest RNAs generated by transcription of SINEs as well as of 7SK and 7SL genes. Functional elements required for regulating gene expression rate are all found within less than 500 base pairs relative to the TSS. In contrast, enhancer elements are often distributed over tens or hundreds of kilobases in the case of Pol II genes (reviewed in Dieci et al. (2007), Dumay-Odelot et al. (2014)). The Pol III gene regulatory elements include DNA sequences showing enhancer-like features to regulate transcription levels ([PSE]; [DSE]; B-box) and promoter elements (TATA-like box; A-box) that are required for positioning Pol III at the TSS. At the protein level, the functional entities for DNA recognition and polymerase recruitment described in the Pol II transcription system are also found in Pol III transcription. However, some of the functions performed by general transcription factors in the Pol II system appear to be fully integrated into the Pol III enzyme. Indeed, structural and/or functional similarities with Pol II transcription factors were identified in four of the 17 Pol III subunits. TFIIE-comparable structure-function modules were described in the POLR3C (RPC3) and POLR3F (RPC6) subunits (Lefevre et al., 2011; Blombach et al., 2015; Ramsay et al., 2020; Girbig et al., 2021; Li G. et al., 2021) and similarities to TFIIF were found in the POLR3D (RPC4) and POLR3E (RPC5) subunits (Ramsay et al., 2020; Li L. et al., 2021; Wang et al., 2021; Girbig et al., 2021 and references therein; Figure 1). The complex composition of Pol III by 17 subunits can probably be explained by the structure of the genes that are transcribed by this enzyme. These genes are short and often possess gene internal or hybrid promoters (type 1, type 2 without or with regulatory elements upstream the TSS; Figure 2), which are bound by TFIIIC. As a consequence, TFIIIC needs to be removed to allow for Pol III to progress through the gene during transcription. Additional transcription factors might complicate this task. Therefore, stable integration of TFIIE- and TFIIF-like activities into polymerase subunits may contribute to a highly efficient transcription mode deemed “facilitated reinitiation” (Dieci and Sentenac, 1996; Dieci et al., 2002; Ferrari et al., 2004).

Furthermore, activities attributed to either general transcription factors or transcriptional activators in the Pol II system were found in the same protein or protein complex in the Pol III transcription system. On the one hand, *Xenopus laevis* TFIIIA is indispensable to recognize the 5S type 1 promoter (A- and C-box; Figure 2A), which corresponds to a function attributed to a general transcription factor. On the other hand, it possesses a transcriptional activation domain that is not needed for promoter recognition but is essential for transcriptional activation. Without this 14-amino acid domain at the C-terminus of *Xenopus laevis* TFIIIA, 5S rRNA gene transcription is undetectable *in vitro* (Mao and Darby, 1993). Furthermore, it has been reported that the three most C-terminal of the nine TFIIIA zinc fingers also exert a higher influence on transcription rate than on promoter recognition (Del Rio and Setzer, 1993).

The functions of TFIIIC in promoter recognition and transcriptional activation can also be separated. In the yeast *Saccharomyces cerevisiae*, the type 2 promoter of the U6 gene is localized partly within the transcribed region and partly downstream of the RNA coding sequence. The intragenic A-box is involved in start site selection along with the TATA box, whereas the B-box is required downstream of the transcription termination site for transcriptional activation *in vivo*. Importantly, the orientation of the B-box is irrelevant for transcriptional activation, demonstrating characteristics of a typical enhancer element (Gabrielsen and Sentenac, 1991; Burnol et al., 1993). Since *S. cerevisiae* TFIIIC is composed of two submodules, τA and τB, which bind to A-Box and B-Box, respectively, general transcription factor activity can be assigned to τA and activator-like functions to τB (Baker et al., 1987; Vorländer et al., 2020). It has not been determined whether this separation of transcriptional activities also holds true for TFIIIC in higher eukaryotes.

In summary, it should be noted that the compact Pol III transcription system combines within the same proteins or protein complexes the functions that are either attributed to transcriptional activators or to general transcription factors in the Pol II system. In addition, functions of some general Pol II transcription factors have been intrinsically integrated into Pol III.

The compact organization of Pol III genes and their requirement for a small number of regulatory DNA elements, the limited number of Pol III transcription factors as well as the major functions that were described for the most prominent Pol III-transcribed RNAs (tRNAs and 5S rRNA in translation; U6 snRNA in mRNA splicing) led to the suggestion that Pol III transcription fulfills primarily housekeeping functions in cells. These housekeeping functions supporting RNAs are thought to be essential for cell survival, but it was long assumed that their expression did not require any regulation since they are thought to be provided in excess compared to the needs of cells (reviewed in Dieci et al. (2007)). The identification of mutations in genes encoding Pol III subunits that lead to the development of hypomorphic diseases, including neurodegenerative disorders, could be considered to result from a failure of these housekeeping functions. Alternatively, it could indicate that Pol III transcription or its RNA products require cell type-specific regulation, which could explain why cells of the central nervous system are more vulnerable than other cells in the body to a loss of homeostasis upon Pol III mutations.

In addition to housekeeping functions, several discoveries unraveled central roles of Pol III in regulatory rather than simply supportive cellular functions. It has become clear that Pol III transcription cannot be separated from the regulation of hypermorphic processes such as tumorigenesis (not discussed here but exemplified or reviewed in White (2008), Pavon-Eternod et al. (2009), Dumay-Odelot et al. (2010), Goodarzi et al. (2016), Durrieu-Gaillard et al. (2018), Gouge and Vannini (2018), Petrie et al. (2019), Yang et al. (2020), Yeganeh and Hernandez (2020)). Moreover, Pol III transcription is an integral part of innate immune defense mechanisms (Carter-Timofte et al., 2018b).

## RNA Polymerase III and Diseases

### Hypomyelinating Leukodystrophy and Related Disorders

Biallelic pathogenic variants in genes encoding Pol III subunits cause a wide spectrum of neurodegenerative disorders.

Within the past decade, it was discovered that biallelic pathogenic variants in six genes encoding subunits of Pol III cause a spectrum of rare inherited disorders (Bernard et al., 2011; Saitsu et al., 2011; Tetreault et al., 2011; Thiffault et al., 2015; Dorboz et al., 2018; Franca et al., 2019; Beauregard-Lacroix et al., 2020; Terhal et al., 2020). The hypomyelinating leukodystrophy (HLD) called 4H leukodystrophy was the first and most commonly identified disease associated with Pol III dysfunction. Since then, the phenotypic spectrum has continued to widen to include both milder and more severe neurodegenerative diseases, as well as rare forms of premature aging or impaired puberty, and gave rise to the name POLR3-related disorders. In this section, we will first describe the major clinical and genetic characteristics of each disease entity within this spectrum. Next, we will review the current state of knowledge on the possible pathogenic mechanisms underlying these diseases. It is important to note that the phenotypic heterogeneity of POLR3-related disorders suggests that several distinct disease mechanisms are likely responsible for different clinical manifestations, perhaps by affecting different functional domains of the enzyme and/or in a cell-type specific manner.

**FIGURE 3:**
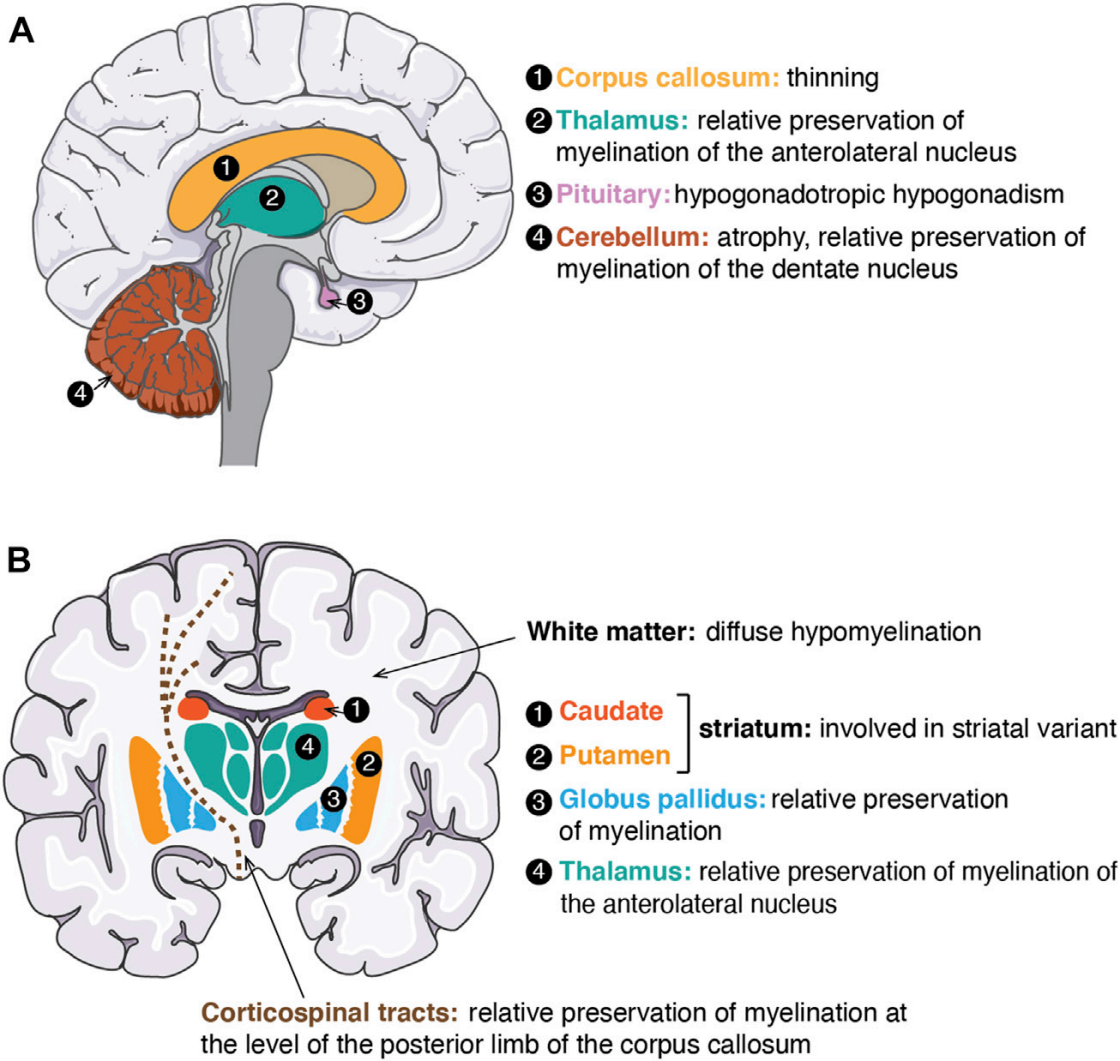
Neuro-anatomical structures affected or for which myelination is preserved in POLR3-related disorders. **(A)** Schematic of a sagittal view of the human brain. Structures involved/preserved in POLR3-HLD are depicted in distinct colours and labeled with a number. On the right side, the names of anatomical structures corresponding to each number are shown in the same colour as the structure, followed by a description of how the structure is affected/preserved in POLR3-HLD. **(B)** Schematic of a coronal view of the human brain. Structures involved/preserved in the striatal variant of POLR3-related disorders (caudate and putamen) or in POLR3-HLD (other structures) are shown in distinct colours and labeled with a number. The legend on the right side follows the same description as in **(A)**. White matter (in white on the brain schematic) is indicated by an arrow. Corticospinal tracts are displayed as brown dashed lines. The figure was adapted from images available on https://smart.servier.com.

Leukodystrophies are a group of genetically determined diseases of the cerebral white matter (Vanderver et al., 2015; van der Knaap and Bugiani, 2017). They are divided according to their Magnetic Resonance Imaging (MRI) characteristics and whether the pathophysiological mechanism is thought to be a lack of myelin deposition during development (hypomyelinating) or alteration of myelin homeostasis (i.e. demyelination or other mechanisms) (Schiffmann and van der Knaap, 2009; Steenweg et al., 2010; Parikh et al., 2015). POLR3-HLD is now recognized as one of the most common hypomyelinating leukodystrophies (Schmidt et al., 2020). It is also referred to as 4H leukodystrophy, where the 4Hs represent the cardinal clinical features: Hypomyelination, Hypodontia and Hypogonadotropic Hypogonadism (Bernard and Vanderver, 1993; Vanderver et al., 2015). Clinical manifestations and anatomical structures involved in POLR3-HLD are shown and described in Table 1 and Figure 3. From 2003 until 2011, before the discovery of the first causal genes, five distinct disorders were described that are now recognized as phenotypes of POLR3-HLD: leukodystrophy with oligodontia (Atrouni et al., 2003), 4H syndrome (Timmons et al., 2006), ataxia, delayed dentition and hypomyelination (Wolf et al., 2007), hypomyelination with cerebellar atrophy and hypoplasia of the corpus callosum (Sasaki et al., 2009), and tremor ataxia with central hypomyelination (Bernard et al., 2010; Tétreault et al., 2012).

**TABLE 1:** Description of the main clinical manifestations observed in POLR3-related disorders and anatomical structures involved.

Clinical manifestation	Description	Anatomical structure(s) involved
Neurological manifestations		
Cerebellar		Cerebellum and/or cerebellar tracts
Gait ataxia	Incoordination or clumsiness of gait	
Dysmetria	Incoordination in limb movements	
Dysarthria	Slurred and dysrhythmic speech	
Dysphagia	Difficulty swallowing	
Pyramidal tract signs		Corticospinal tracts
Spasticity	Velocity dependent increased muscle tone	
Brisk reflexes	Abnormally brisk stretch reflexes	
Extrapyramidal signs		Basal ganglia (striatum) connections
Dystonia	Movement disorder characterized by involuntary contractions of muscles leading to abnormal postures, twisting movements and/or tremor	
Non-neurological manifestations		
Hypodontia	Developmental absence of tooth/teeth	Teeth
Hypogonadotropic hypogonadism	Delayed/absent/arrested puberty, growth hormone deficiency	Pituitary gland
Endosteal sclerosis	Sclerosis of the endosteum (layer of vascular connective tissue lining the medullary cavities of bone)	Bone
Progeroid appearance, progeria	Aged appearance, premature aging	N/A

Our group and others identified the first and most commonly mutated genes in POLR3-HLD, *POLR3A* and *POLR3B* (Bernard et al., 2011; Saitsu et al., 2011; Tétreault et al., 2011; Daoud et al., 2013)*.* We later described a third, less commonly mutated gene, *POLR1C* (Thiffault et al., 2015)*,* and also *POLR3K* as a fourth and rare causal gene (Dorboz et al., 2018). Patients with POLR3-HLD typically present in early childhood with motor delay or regression (Vanderver et al., 2013). POLR3-HLD primarily affects the central nervous system (CNS). The predominant neurological features are cerebellar (i.e. gait ataxia, dysmetria, dysarthria), followed by pyramidal (i.e. spasticity, brisk reflexes, etc., often affecting predominantly the lower extremities), extrapyramidal (especially dystonia) (Osterman et al., 2012; Al Yazidi et al., 2019) and cognitive (i.e. intellectual disability and/or cognitive regression) features (Wolf et al., 2014a; Gauquelin et al., 2019). The disease is progressive or neurodegenerative, resulting in progressive motor impairment leading to loss of ambulation, progressive dysarthria leading to loss of speech, progressive dysphagia leading to tube feeding dependency and eventually to premature death. Non-neurological features are typically but not universally present (Wolf et al., 2014a; Gauquelin et al., 2019) and include myopia, typically progressive over several years, dental abnormalities (e.g. hypodontia, oligodontia, delayed or abnormal pattern of tooth eruption, natal tooth/teeth, etc.) (Wolff et al., 2010) and endocrine abnormalities, typically but not exclusively, hypogonadotropic hypogonadism leading to arrested or absence of puberty (Potic et al., 2012, 2015; Pelletier et al., 2021). The MRI of patients with a typical POLR3-HLD is characterized by a specific and recognizable pattern of hypomyelination (Schiffmann and van der Knaap, 2009; Steenweg et al., 2010), with relative preservation of the myelination of certain structures (i.e. dentate nucleus, optic radiations, anterolateral nucleus of the thalamus, globus pallidus, and in some cases, of the corticospinal tracts at the level of the posterior limb of the internal capsule), as shown in Figure 3. Atrophy of the cerebellum and thinning of the corpus callosum are commonly seen (Steenweg et al., 2010; La Piana et al., 2014; Wolf et al., 2014a) (Figure 3). Patients with POLR3-HLD require multidisciplinary care for their complex medical needs (Adang et al., 2017).

Although POLR3-HLD is the most common form of POLR3-related disorders, there is a spectrum of several disease entities caused by mutations in genes encoding Pol III subunits. At the most severe end of the spectrum are patients with a specific combination of *POLR3A* variants, leading to the severe striatal variant, which is clinically and radiologically distinct from the typical POLR3-HLD, with prominent involvement of the basal ganglia (Figure 3). These patients present at 2–3 months of life with developmental delay and regression and severe dysphagia (Perrier et al., 2020a; Harting et al., 2020; Hiraide et al., 2020). They develop respiratory failure and a significant proportion of them become bedridden and/or die during early childhood.

Another form of POLR3-related disorder is the Wiedemann-Rautenstrauch syndrome (WRS), caused by specific combinations of *POLR3A* mutations (Jay et al., 2016; Paolacci et al., 2018; Wambach et al., 2018). These patients present intrauterine growth retardation and post-natal failure to thrive, together with a progeroid appearance. They also typically have a triangular face, convex or pinched nose, a small mouth, sparse hair and lipodystrophy. Their fontanelles may be enlarged and pseudohydrocephalus with prominent scalp veins may be observed. Dental abnormalities reminiscent of POLR3-HLD can be seen, including natal tooth/teeth. Some of these patients have both WRS and POLR3-HLD.

At the other end of the spectrum are mild presentations. This category includes patients homozygous for the common *POLR3B* mutation c.1568T>A (p.Val523Glu), who may remain asymptomatic or paucisymptomatic until adulthood and even late adulthood (DeGasperis et al., 2020; Perrier et al., 2020a; Verberne et al., 2020; Wolf et al., 2014a). Also in this category are the patients with the mild striatal variant, without hypomyelination but with basal ganglia involvement on the brain MRI (Figure 3), who carry a very specific combination of *POLR3A* splice site variants (Azmanov et al., 2016). Another group presenting a milder presentation include patients with spastic ataxia and spastic paraparesis without hypomyelination (La Piana et al., 2016; Minnerop et al., 2017; Gauquelin et al., 2018; Rydning et al., 2019). Some patients with biallelic pathogenic variants in *POLR3B* can present mainly or uniquely with endocrine manifestations (Richards et al., 2017). Patients with mutations in *POLR3B* can present with cerebellar involvement and the bone manifestation of endosteal sclerosis (Ghoumid et al., 2017). Most recently, specific *de novo* pathogenic variants in *POLR3B* have been associated with ataxia, spasticity and demyelinating neuropathy without CNS hypomyelination (Djordjevic et al., 2021). Patients with biallelic variants in *POLR3GL* can present with endosteal hyperostosis and oligodontia (Terhal et al., 2020) or WRS (Beauregard-Lacroix et al., 2020). Finally, a homozygous variant in *POLR3H* has been associated with primary ovarian insufficiency (Franca et al., 2019).

Interestingly, although not technically a part of POLR3-related disorders, mutations in *BRF1*, encoding a subunit of the Pol III transcription factor TFIIIB-β, cause a cerebellar-facial-dental syndrome with clinical overlap with POLR3-related disorders (Borck et al., 2015; Jee et al., 2017), emphasizing the vulnerability of these tissues to Pol III dysfunction.

Although POLR3-related disorders, and more specifically POLR3-HLD, have been extensively characterized at the clinical and genetic levels, the functional consequences of the various mutations in genes encoding Pol III subunits are not well understood. To this date, no curative treatment is available and supportive care is the standard. Understanding the pathophysiology of these diseases will be key in order to develop therapies that can be tested in the pre-clinical setting and eventually translated to the clinic (Perrier et al., 2020b). Specifically, it remains enigmatic how mutations in a ubiquitously expressed and essential enzyme such as Pol III lead to disorders with clinical features that are largely restricted to the CNS and a few other tissues, all of which originate from neural crest cells. The pathophysiological mechanisms underlying such a wide spectrum of phenotypes are also unclear. Importantly, depending on the phenotype, different CNS cell types are affected, including oligodendrocytes, the cells that produce myelin, several populations of neurons, and/or their respective progenitor cells (Figure 3 and Table 1). Hypomyelination in POLR3-HLD is thought to result from oligodendrocyte dysfunction, but cerebellar atrophy indicative of cerebellar neuron involvement is also observed (Vanderver et al., 2013; Wolf et al., 2014b). The other neurodegenerative phenotypes are postulated to result from abnormalities of cerebellar neurons (spastic ataxia) or of the basal ganglia, or brain atrophy (striatal variants) (Minnerop et al., 2017; Perrier et al., 2020a). Thus, identifying one unified disease mechanism for all POLR3-related disorders is not expected. Instead, distinct cell types may be differently affected by Pol III dysfunction, leading to a mechanistic diversity that would reflect the genetic and phenotypic heterogeneity of POLR3-related disorders.

**FIGURE 4:**
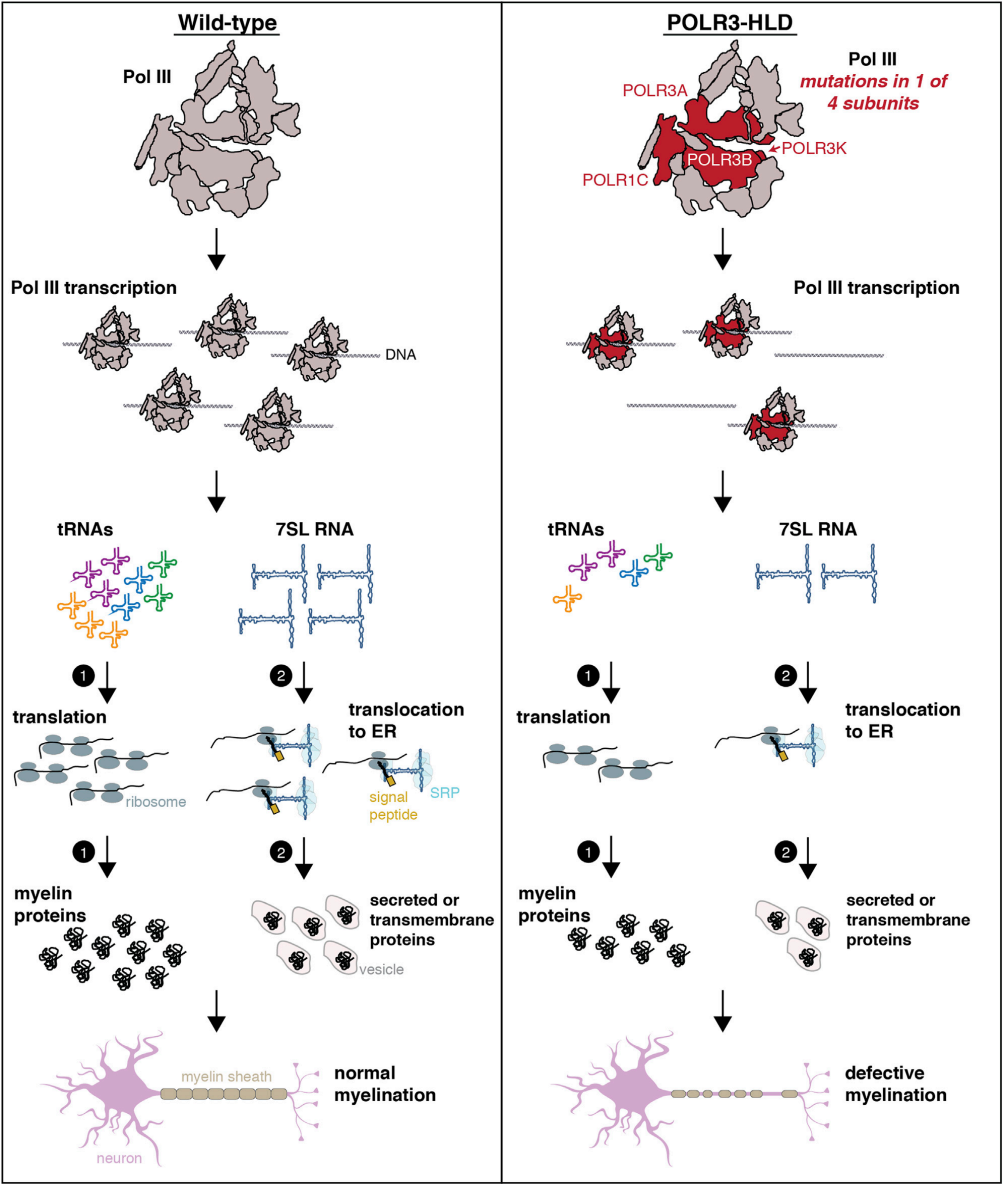
Schematic representing possible mechanisms underlying POLR3-HLD. In wild-type conditions (healthy individuals), Pol III synthesizes small ncRNAs that play essential roles in housekeeping processes such as translation and co-translational targeting of nascent peptides, which are necessary for the production of myelin. In individuals with POLR3-HLD, it is hypothesized that mutations in Pol III subunits (POLR3A, POLR3B, POLR1C or POLR3K) result in reduced Pol III transcription and decreased levels of Pol III transcripts. The “tRNA-centric” hypothesis postulates that lower levels of tRNAs (either globally, for specific anticodons or for specific isodecoders) will impact translation and synthesis of proteins that are essential for myelination. Alternatively or in addition, reduced levels of other Pol III transcripts may contribute to POLR3-HLD pathogenesis through suboptimal performance of their respective functions that will particularly affect oligodendrocytes and/or neurons. An example is shown for 7SL RNA, where reduced levels of this ncRNA could impair translocation of secreted or transmembrane proteins to the ER, which could impact production of myelin. The schematic of the neuron was adapted from images available on https://smart.servier.com.

There are two main pathophysiological hypotheses in the field, which are not mutually exclusive (Figure 4). Specifically for hypomyelination in POLR3-HLD, the first hypothesis states that hypofunctional Pol III, secondary to mutations in genes encoding Pol III subunits, leads to reduced levels of tRNA (either globally or of specific anticodons or isodecoders) and/or other small non-coding RNA (ncRNA) important for translation in a critical developmental period such as myelination. Since most of the myelination process occurs in a relatively short period of time, i.e. in the first 2 years of life in humans, it is thought that oligodendrocytes, the cells responsible for myelin production in the CNS, are more susceptible to a hypofunctional Pol III or reduced translation capacity due to the high metabolic requirements of producing myelin. Indeed, oligodendrocytes must produce a large amount of lipids and myelin-specific proteins to deposit on axons during myelination (Pfeiffer et al., 1993; Anitei and Pfeiffer, 2006). A hypomorphic Pol III would therefore impair global protein production during this critical developmental window leading to improper formation of myelin, ultimately causing the hypomyelination phenotype (Lin and Popko, 2009; Fröhlich et al., 2018; Torrent et al., 2018). This hypothesis is supported by the recent description of several hypomyelinating disorders caused by mutations in genes important for protein translation such as those encoding for tRNA-aminoacyl synthetases, including *DARS1, EPRS1* and *RARS1,* amongst others (Taft et al., 2013; Wolf et al., 2014a; Mendes et al., 2018, 2020). This raises the possibility that certain codons are particularly important for proper CNS function, and that reduced availability of the corresponding aminoacyl-tRNA through Pol III or tRNA-synthetase mutations is particularly detrimental to the CNS. Moreover, the CNS may have a lower threshold than other tissues for tolerating hypofunction of these enzymes. Another supportive element is that the brain MRI of patients with hypomyelination that carry mutations in genes encoding Pol III subunits show an arrested myelination, with myelination of the early myelinating structures, which are the smallest in size, but not the rest of the brain, suggesting that myelination began properly but could not be completed, perhaps due to impaired protein synthesis.

The second hypothesis, which can be generalized to all POLR3-related disorders, states that Pol III hypofunction leads to decreased levels of specific Pol III transcripts involved in transcription, RNA processing and/or translation, which preferentially perturbs the expression and/or translation of mRNAs that are essential for the development, survival and function of oligodendrocytes and/or neurons (Tétreault et al., 2011; Thiffault et al., 2015; Azmanov et al., 2016; Minnerop et al., 2017; Choquet et al., 2019a). An example of this hypothesis is shown in Figure 4 for 7SL RNA, but it can be extended to any Pol III transcript and their specific function. These two non-mutually exclusive hypotheses may both contribute to the distinct phenotypes observed in POLR3-related disorders, with perturbation of different Pol III transcripts and their downstream functions having cell type- or temporal-specific effects.

Recent efforts to better understand the pathophysiological mechanisms of POLR3-related disorders have focused on three main areas: the impact of Pol III subunit mutations on biogenesis of the Pol III complex; the downstream consequences of mutations on the Pol III transcriptome; and the development of animal models of the disease.

### Impact of Pol III Subunit Mutations on Enzyme Biogenesis

The recessive mode of inheritance and the nature of most disease-causing mutations (missense, splice site, truncating) in genes encoding Pol III subunits suggests a hypomorphic disease mechanism, either through decreased protein abundance or because of abnormal interactions of the mutated subunit with other subunits, with DNA or with RNA (Bernard et al., 2011). Given the genetic and phenotypic heterogeneity of POLR3-related disorders, distinct mutations may have different effects on mRNA or protein stability or on Pol III function itself, leading to different phenotypes or modulating disease severity. Decreased levels of mRNA or protein encoded by the mutated gene have been observed in fibroblasts, blood, white matter or cortex of individuals with *POLR3A* or *POLR3GL* mutations (Bernard et al., 2011; Azmanov et al., 2016; Minnerop et al., 2017; Perrier et al., 2020a; Báez-Becerra et al., 2020; Beauregard-Lacroix et al., 2020), the majority of which carried a truncating mutation on one allele. While mRNA or protein levels have not been examined extensively in individuals with missense mutations, two reports suggest that they are not always altered. First, mice homozygous for the *Polr3a* c.2015G>A (p.Gly672Glu) mutation had normal POLR3A protein levels (Choquet et al., 2017). Second, *POLR3K* mRNA levels were unchanged in individuals carrying missense mutations in this gene (Dorboz et al., 2018).

Missense mutations in *POLR3A*, *POLR3B* and *POLR1C* causing POLR3-HLD are located throughout the three genes without clear hotspots (Wolf et al., 2014a; Gauquelin et al., 2019; Ramsay et al., 2020; Li G. et al., 2021; Girbig et al., 2021) and affect most major structural regions (Arimbasseri and Maraia, 2016). Prior to the publication of the first Pol III yeast structures (Abascal-Palacios et al., 2018; Vorländer et al., 2018), the potential impact of *POLR3A* and *POLR3B* HLD mutations was predicted *in silico* by extrapolating them onto the yeast Pol II structure. This suggested that most of these amino acid changes would impair the interaction with other Pol III subunits or with the DNA template (Bernard et al., 2011; Saitsu et al., 2011; Tétreault et al., 2011; Wolf et al., 2014a). Similarly, the only reported *POLR3K* mutation was predicted using the yeast Pol III structure to decrease protein stability and to impair the interaction between POLR3K and POLR3B (Dorboz et al., 2018). Recently, the tridimensional structure of the human Pol III was resolved by cryogenic electron microscopy (Ramsay et al., 2020; Li L. et al., 2021; Girbig et al., 2021; Wang et al., 2021). First, Ramsay et al., mapped 47 POLR3-HLD mutations in *POLR3A, POLR3B* and *POLR1C* onto the Pol III structure, which revealed that they cluster in regions at the interface of several subunits and are predicted to disrupt these interfaces, consistent with the earlier predictions made using the yeast Pol II structure. Second, Girbig et al., investigated 110 point mutations found in patients with POLR3-related disorders and classified them into four types, showing that the majority of POLR3-HLD mutations are predicted to disturb the core of a given subunit (Type I) or are located at the interface between subunits and have the potential to impair complex assembly (Type III), while a smaller number affect functional elements such as the bridge helix or the trigger loop (Type II). Li et al., mapped several mutations in Treacher Collins syndrome (TCS), WRS and POLR3-HLD. They suggest that these mutations may impair complex integrity or enzymatic activity.

**FIGURE 5:**
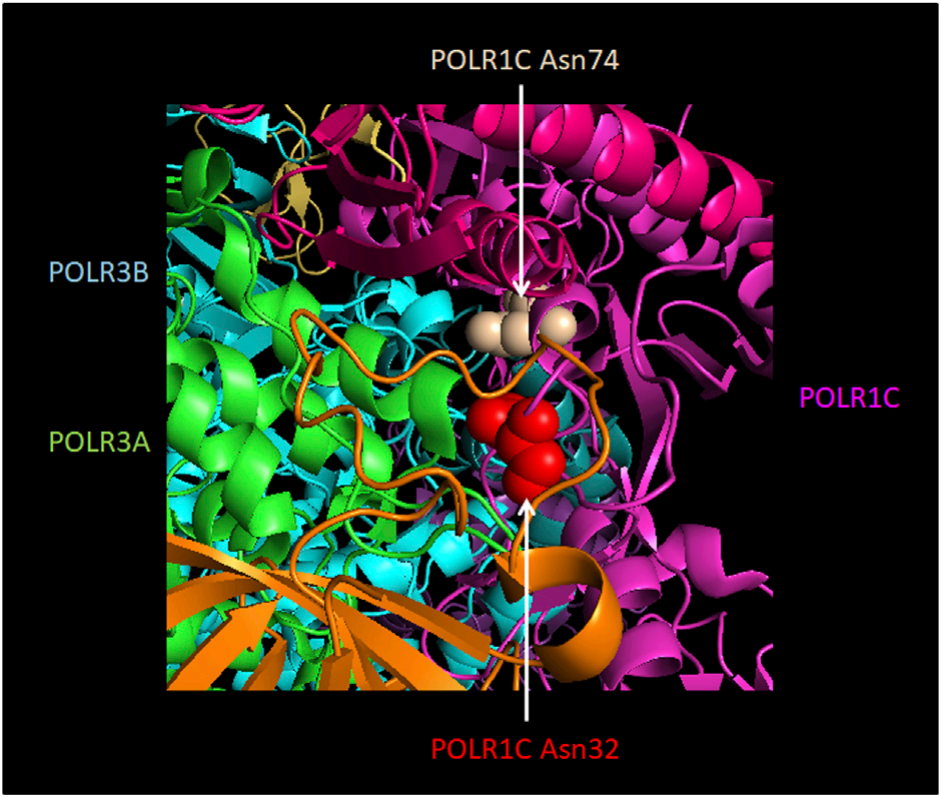
Localization of POLR1C Asn32Ile and Asn74Ser mutations that are associated with POLR3-HLD. Amino acids in POLR1C which cause an assembly defect in Pol III (Thiffault et al., 2015) upon mutation are shown in red (POLR1C Asn32) and in ochre (POLR1C Asn74). They are localized at the interface with Pol III subunits POLR3A (green) and POLR3B (turquoise). The remainder of POLR1C is colored in pink. The Figure was modified from PDB 7AE3 by employing Pymol.

**FIGURE 6:**
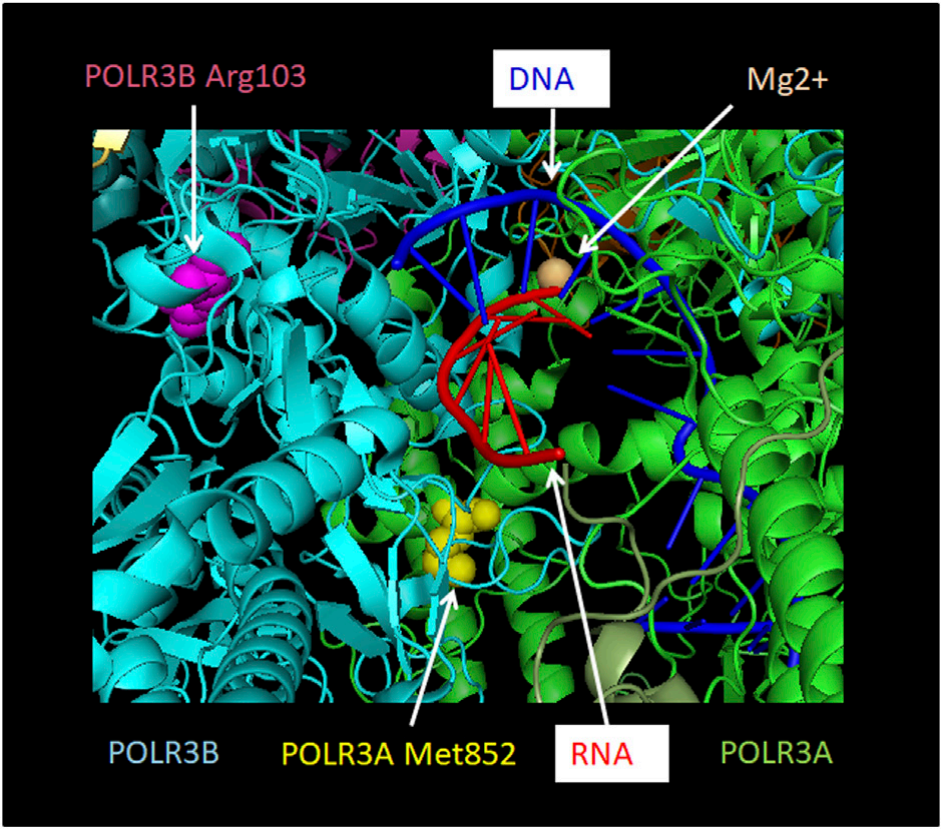
Localization of POLR3A Met852Val and POLR3B Arg103His mutations relative to the active site. POLR3A is shown in green and POLR3B in turquoise. Pol III mutations described in the text that are found close to the active site and which may thus affect catalytic activity are depicted. Methionine 852 of POLR3A as part of the bridge helix is highlighted as a sphere in yellow. Arginine 103 of POLR3B is highlighted as a sphere in magenta. RNA is shown in red and DNA in blue. The Mg2+ ion of the active site is shown as a sphere in light orange. The Figure was modified from PDB 7AE3 by employing Pymol.

In addition to these *in silico* predictions, the effect of some mutations on Pol III complex assembly has been assessed experimentally (Thiffault et al., 2015; Choquet et al., 2017, 2019a, 2019b; Djordjevic et al., 2021). In this series of experiments, the wild-type or mutated Pol III subunit of interest was exogenously expressed with a FLAG tag, allowing subsequent affinity purification and shotgun proteomics to identify interacting partners. The first such study focused on POLR1C and demonstrated that two HLD-causing mutant versions of this subunit (Asn32Ile and Asn74Ser) pulled down significantly lower levels of other Pol III subunits compared to the wild-type subunit, indicating a defect in Pol III complex assembly (Thiffault et al., 2015). This was supported by immunofluorescence data showing that while wild-type POLR1C was predominantly present in the nucleus, mutated POLR1C variants tended to accumulate in the cytoplasm, where Pol III biogenesis takes place. Consistent with these results, mapping of these residues onto the human Pol III structures suggested a function in mediating interactions with POLR3A and POLR3B (Ramsay et al., 2020; Figure 5) and postulated that they would impair complex assembly (Girbig et al., 2021). Only one POLR3-HLD-causing *POLR3B* mutation (Arg103His; Figure 6) was assayed in a similar manner and was also found to severely impair Pol III complex assembly (Choquet et al., 2019b), while it was predicted to disrupt the core of the subunit in structural studies (Girbig et al., 2021). In contrast, two *POLR3A* mutations, Gly672Glu and Met852Val (Figure 6), had no impact on Pol III biogenesis using the same assay (Choquet et al., 2017; 2019a), although they were predicted to impact assembly with POLR2H and to destabilize the POLR3A/POLR3B interface, respectively, in one Pol III structural study (Ramsay et al., 2020), while they were classified as disrupting the core of the subunit and impacting functional elements, respectively, in the second structural study (Girbig et al., 2021). Indeed, POLR3A Met852Val is localized in the vicinity of the bridge helix (Figure 6), so it could impair interaction with DNA or transcription itself rather than enzyme assembly (Bernard et al., 2011). Moreover, it is worth noting that POLR3A Gly672Glu can cause a relatively mild phenotype in human individuals (Bernard et al., 2011; Wolf et al., 2014a) and does not lead to neurological abnormalities in mice (Choquet et al., 2017) (see below), which may be due in part to the correct biogenesis and nuclear import of Pol III when this mutation is present.

Using the same experimental system, recently described *POLR3B de novo* heterozygous mutations, which cause a distinct phenotype and are thought to act through a dominant negative mechanism, were found to disrupt the interaction of POLR3B with only one or two Pol III subunits instead of causing an assembly defect of the entire complex (Djordjevic et al., 2021), as was seen for the *POLR1C* and *POLR3B* mutations above. Mapping of these mutations onto the yeast Pol III structure suggests that they are involved in DNA melting or transcription itself (Djordjevic et al., 2021). This indicates that the structural and mechanistic impact of various Pol III pathogenic variants may underlie some of the phenotypic differences observed in patients.

### Impact of Pol III Subunit Mutations on the Pol III Transcriptome

Decreased protein abundance, defective Pol III biogenesis and nuclear import or impaired interaction with DNA are all hypothesized to lead to the common outcome of reduced Pol III transcriptional output (Figure 4), resulting in some shared clinical symptoms despite differences in the structural and mechanistic consequences of the mutations. Nonetheless, Pol III transcript deficiencies may be different across cell types or as a consequence of different mutations, thus underlying some of the observed phenotypic heterogeneity. Indeed, expression profiling of the Pol III transcriptome in patient cells or disease models by several groups has revealed a complex picture.

As quantification of most Pol III transcripts is challenging due to their small size, post-transcriptional modifications and repetitive nature, measurement of Pol III occupancy on DNA has often been used as a proxy for Pol III transcription levels (Kutter et al., 2011; Canella et al., 2012). ChIP-seq of FLAG-tagged mutated versions of POLR1C showed a global decreased occupancy at all types of Pol III target promoters, consistent with the low nuclear levels of these POLR1C variants (Thiffault et al., 2015). In contrast, no significant differences in Pol III occupancy were observed by ChIP-qPCR of three Pol III-transcribed loci with exogenous POLR3A-Gly672Glu (Choquet et al., 2017) or by ChIP-seq in cell lines carrying an endogenous *POLR3A* Met852Val mutation (Choquet et al., 2019a), suggesting that these POLR3-HLD-causing mutations may directly impact transcription itself rather than Pol III binding to DNA.

While several recent studies have reported decreased levels of some Pol III transcripts as a result of disease-causing mutations, the identity of these transcripts varies from one study to another (Azmanov et al., 2016; Dorboz et al., 2018; Choquet et al., 2019a). Azmanov et al. (2016) were the first to perform a transcriptome-wide characterization of blood cells from patients with the mild striatal variant of POLR3-related disorders and a specific homozygous splice site mutation (c.1771-6C>G) in *POLR3A*. They observed a global but mild decrease in mature tRNA levels, with only seven tRNAs reaching statistical significance, a reduction in 7SL RNA levels and an increase in the levels of 5S rRNA, RNase P RNA (H1), 7SK RNA and RNase MRP RNA (Azmanov et al., 2016). In a second study, targeted analysis of fibroblasts from two HLD patients carrying *POLR3K* mutations found decreased levels of initiator tRNA^Met^ but no change for three other tRNAs, as well as reduced expression of 7SK RNA and a more severe decrease of 7SL and 5S rRNA levels (Dorboz et al., 2018). Third, CRISPR-Cas9 was used to introduce an endogenous HLD *POLR3A* Met852Val mutation in HEK293 cells in compound heterozygosity with a null allele. Transcriptome-wide analysis uncovered a global decrease in precursor tRNA levels, 7SL RNA and the primate-specific neural BC200 RNA, while other transcripts were not affected (Choquet et al., 2019a). BC200 RNA was also downregulated in the oligodendroglial cell line MO3.13 edited with the same genotype and in two small cohorts of HLD patient-derived fibroblasts carrying *POLR3A* mutations (individual mutations listed in Table S5 of Choquet et al., 2019a). The levels of this RNA were not assessed in the two aforementioned studies (Azmanov et al., 2016; Dorboz et al., 2018). Lastly, qRT-PCR analysis of fibroblasts from a WRS patient showed an increase of tRNA-Leu-CAA, a decrease in 7SK RNA and a virtual absence of 5S rRNA (Báez-Becerra et al., 2020).

These four datasets present important differences in terms of pathogenic variants, nature of the variants, associated phenotypes, cell types assayed and experimental approaches, thus it is not surprising that the affected transcripts vary. Nonetheless, some common trends are starting to emerge. Among disorders with predominant CNS manifestations (mild striatal phenotype or HLD) (Azmanov et al., 2016; Dorboz et al., 2018; Choquet et al., 2019a), 7SL RNA stands out as possibly the most commonly affected transcript. However, an earlier report observed that this transcript had comparable levels in fibroblasts from one POLR3-HLD patient and a healthy control (Shimojima et al., 2014). A subset of tRNAs were also downregulated in each of the three studies, including a common decrease in the initiator tRNA^Met^ levels, which reached statistical significance in two out of three datasets. In the study using POLR3A-edited cell lines and two small patient cohorts, BC200 RNA also emerged as a downregulated Pol III transcript.

7SL and BC200 are both transcribed through a hybrid Pol III promoter (Figure 2D), while tRNA genes use a standard type 2 promoter (Figure 2B) (Choquet et al., 2019a). In contrast, the two downregulated transcripts in the WRS patient, 5S rRNA and 7SK RNA, are transcribed through type 1 and 3 promoters, respectively (Báez-Becerra et al., 2020; Figures 2A,C). It is tempting to hypothesize that alterations in the levels of distinct Pol III transcripts or promoter types may be responsible for different phenotypes. However, in fibroblasts from two patients with *POLR3K* HLD-causing mutations (Dorboz et al., 2018), 5S rRNA levels were decreased in both patients and 7SK levels were diminished in one patient, suggesting a more complex picture. Indeed, mutations in *BRF1*, encoding a subunit of the transcription factor TFIIIB-β specific to Pol III type 1 and type 2, as well as some hybrid promoters, cause a cerebellar-facial-dental syndrome. Analysis of the corresponding mutations in yeast showed impaired Pol III transcription of a tRNA gene *in vitro* (Borck et al., 2015). This disorder overlaps phenotypically with POLR3-related disorders but does not include hypomyelination (Borck et al., 2015; Jee et al., 2017). Together with alterations of tRNAs and 7SL RNA in the mild striatal variant (Azmanov et al., 2016), this argues against the hypothesis that perturbation of the transcription of type 2 or hybrid Pol III target genes specifically leads to myelination defects. Furthermore, as type 2 target genes, especially tRNA genes, far outnumber those with type 1 or 3 target genes, it is not unexpected that the most affected genes in these studies would belong to the former group.

Nonetheless, these gene expression studies emphasize that Pol III transcript levels are remarkably resistant to genetic perturbations in the enzyme, since only a proportion of Pol III transcripts are affected, while many show no change. Importantly, the majority of these datasets were obtained from cell types that are not affected in POLR3-related disorders. Pol III mutations may have a much stronger impact on the transcriptome of affected cell types. Consistent with this idea, the c.1909+22G>A mutation that is common in POLR3-related spastic ataxia results in an aberrant POLR3A splice isoform that is present at higher levels in neuroepithelial cells compared to induced pluripotent stem cells (iPSCs) (Minnerop et al., 2017). Although the Pol III transcriptome was not profiled in these cells, it would be interesting to determine if a higher ratio of aberrantly spliced to wild-type isoform results in stronger alterations of Pol III transcript levels.

The top down-regulated transcripts, 7SL RNA, tRNAs and BC200 RNA, are all involved in mRNA translation and protein homeostasis (Dieci et al., 2007). Quantitative proteomics in POLR3A-edited MO3.13 cells uncovered only a small number of deregulated proteins compared to normal cells (Choquet et al., 2019a). However, since this oligodendroglial cell line was established from a tumor (McLaurin et al., 1995), similar experiments in oligodendrocyte precursor cells (OPCs) derived from human iPSCs or in mouse OPCs, along with ribosome profiling or analysis of nascent proteins, would allow to better determine how translation is impacted upon *POLR3A* mutations in oligodendrocytes. Nevertheless, these cells showed decreased expression of Myelin Basic Protein (MBP) mRNA upon differentiation into more mature oligodendroglial cells, indicating that the mild Pol III transcriptome alterations may be sufficient to alter oligodendrocyte differentiation and/or MBP expression (Choquet et al., 2019a). The observation of nucleolar disruption, activation of p53 and premature senescence in WRS fibroblasts (Báez-Becerra et al., 2020) suggests an alternative mechanism for the pathophysiology of this progeroid syndrome that could be associated with the near absence of rRNAs.

Future research will require larger cohorts from each disease entity within POLR3-related disorders to determine which specific Pol III transcripts are affected and to pinpoint phenotype- and cell-type specific disease mechanisms. Moreover, in order to better understand the pathophysiology of POLR3-related disorders in the relevant cell types, animal models of the diseases are required.

### Development of Animal Models of POLR3-Related Disorders

Initial efforts to generate an animal model of POLR3-HLD were not successful. Homozygous knockout of *Polr3a* in mice is embryonic lethal (Choquet et al., 2017), but whole body knock-in (KI) of the French-Canadian founder mutation *Polr3a* c.2015G>A (p.Gly672Glu) did not lead to any neurological or developmental abnormalities in homozygous animals (Choquet et al., 2017). Pol III transcript levels were also normal in the brain of these KI mice. In contrast, homozygosity for the *Polr3b* c.308G>A (p.Arg103His) mutation, which has only been reported in compound heterozygosity with another missense mutation in humans, is embryonic lethal in mice (Choquet et al., 2019b). Interestingly, the drastically different impacts of these two mutations in mice are consistent with the severity of their effect on Pol III biogenesis in human cells (see above) (Choquet et al., 2017; 2019b).

As the *POLR3A* Gly672Glu mutation leads to disease in humans but not in mice, this could suggest that the latter species is less vulnerable to Pol III mutations or that primate-specific transcripts, such as *BC200* RNA, are involved in the pathogenesis of the disease (Choquet et al., 2017). This is also consistent with the observation that mouse models for leukodystrophies tend to have a milder phenotype (Lu et al., 1997; Pujol et al., 2002; Odermatt et al., 2003; Geva et al., 2010; Tress et al., 2011; Fröhlich et al., 2020), which may be due to the lower amount of myelin in mouse brains compared to humans (Fields, 2008; Jakovcevski et al., 2009; Ornelas et al., 2016; Choquet et al., 2017). Thus, two strategies have been attempted to increase the Pol III mutational burden in the hopes that it would lead to a phenotype in mice. First, *Polr3a* ^G672/G672E^ and *Polr3b* ^+/R103H^ were interbred to generate mice with a homozygous mutation in *Polr3a* and a heterozygous mutation in *Polr3b*. However, these mice did not display neurological abnormalities or alterations in Pol III transcript levels (Choquet et al., 2019b).

The second approach was more successful and generated the first mouse model demonstrating hypomyelination as seen in POLR3-HLD, but with a very mild phenotype and absent motor features (pre-print on https://www.biorxiv.org/content/10.1101/2020.12.09.418657v2, currently under peer review at the time this manuscript is written) (Merheb et al., 2021). To achieve this, the Willis laboratory first screened a panel of *POLR3A* HLD mutations by introducing them in the *S. cerevisiae* orthologous gene, *Rpc160*, focusing on a cluster of mutations in the pore region of Pol III, which included Gly672Glu (Moir et al., 2021). Double mutants were also generated by combining Gly686Glu (corresponding to Gly672Glu in humans) with every other mutation in the pore region. Individually, none of these mutations impaired growth, Pol III transcription or mature Pol III transcript levels in *S. cerevisiae*. However, the double mutants displayed phenotypes ranging from wild type to lethal as well as various sensitivity levels to high or cold temperatures. The authors focused on one double mutant carrying the adjacent Tyr685Lys and Gly686Glu mutations, which had an intermediate growth defect and displayed temperature sensitivity. They observed decreased levels of a subset of Pol III transcripts in this mutant (RNAse P RNA [*RPR1*] and small nucleolar RNA 52 [*SNR52*]), while other RNAs, notably those encoding 7SL RNA and 5S rRNAs, were not affected. *In vitro* transcription experiments demonstrated a defect in both factor-independent and factor-dependent transcription for genes representative of the yeast Pol III transcriptome in this double mutant (Moir et al., 2021). Next, the authors generated mice with the corresponding human double allele Trp671Arg/Gly672Glu (Merheb et al., 2021). Since homozygosity for the whole-body KI of this allele was embryonic lethal, a conditional KI mouse was engineered using an Olig2-Cre driver, directing expression of the mutant allele throughout the oligodendrocyte lineage and in a subset of other CNS cells (Merheb et al., 2021). Homozygous conditional KI mice displayed growth defects, neurobehavioral deficits and impaired myelination, myelin integrity and oligodendrogliogenesis. Although the mouse did not display a motor phenotype compatible with POLR3-HLD, it did show mild neurobehavioral features, and myelin defects reminiscent of HLD. Thus, the Trp671Arg/Gly672Glu KI mouse is the first animal model of POLR3-HLD that recapitulates some of the pathological features of the disease. This model can now be used to better understand the relationship between impaired Pol III function and myelin deficits.

A handful of other animal models with mutations in Pol III subunits have also been engineered. A mutation in *POLR3H* was recently found to cause primary ovarian failure (Franca et al., 2019) and this phenotype was well-recapitulated in whole-body knock-in mice homozygous for the *POLR3H* Asp50Gly mutation (Franca et al., 2019). In zebrafish, a splice site mutation causing the deletion of 41 amino acids in the Polr3b protein led to defects in the development of the intestine, intestinal epithelium and exocrine pancreas (Yee et al., 2007). This mutation impacted the interaction of Polr3b with Polr3k in yeast, and overexpression of *Polr3k* cDNA in zebrafish partially rescued the exocrine pancreas defects. Moreover, conditional deletion of *Polr3b* exon 10 in the mouse intestinal epithelium also led to reduced survival and growth, defective crypt development and increased apoptosis (Kieckhaefer et al., 2016). Interestingly, HLD patients with *POLR3K* mutations present severe digestive dysfunctions that are not typically observed in individuals with mutations in other Pol III subunits (Dorboz et al., 2018). Both patients with biallelic pathogenic variants in *POLR3K* as well as the zebrafish model displayed decreased levels of 7SL RNA, suggesting that the interaction between *POLR3B* and *POLR3K* may be particularly important for transcription of the 7SL RNA gene. Without normal levels of 7SL RNA, its function in protein secretion may be impaired, which could be especially detrimental to normal gut function. The future generation of animal models with a range of mutation types in different Pol III subunits will hopefully help to delineate genotype-phenotype correlations and provide a better understanding of the tissue- and cell type-specific manifestations of POLR3-related disorders. When possible, direct modulation of candidate Pol III transcript (e.g. 7SL RNA) levels in animal models would also help understand the developmental and tissue-specific consequences of their depletion.

As described above, possible reasons for tissue-specific differences may reside in particular dependencies of individual cell types on Pol III transcription products. Below, we will focus on the major Pol III-transcribed RNAs that have shown altered expression in cells carrying mutations in genes associated with POLR3-related disorders. We will describe characteristics of tRNAs, 7SL RNA and BC200 RNA.

## Function of Pol Iii Transcripts and Role in Polr3-Related Disorders

### Expression and Functions of tRNAs

tRNAs are short (76–90 nucleotides) non-coding RNAs that act as essential adapters during mRNA translation. Each tRNA is loaded at their 3’ end with the amino acid corresponding to its anticodon by cytoplasmic aminoacyl tRNA synthetases (aaRS1). tRNAs allow decoding of the genetic code by recognizing cognate codons in translating mRNA and providing the corresponding amino acid for addition to the nascent peptide (reviewed in Lant et al. (2019)). Of the >600 putative tRNA genes in human, approximately 300–400 are expressed in a given human cell (Canella et al., 2010; Oler et al., 2010; Gogakos et al., 2017), resulting in multiple expressed genes with minor sequence differences encoding tRNAs with the same anticodon (isodecoders; Pan, 2018). Sequence changes or imbalanced expression of tRNAs can lead to deregulated translation (reviewed in Lant et al. (2019); Kapur et al. (2020)).

Several studies have shown that pools of expressed tRNA isodecoders vary by cell type and cell state, suggesting that certain isodecoders are more important in specific contexts and that their dysregulation could impair cellular homeostasis. Indeed, distinct pools of tRNAs are expressed between proliferating and differentiating cells (Gingold et al., 2014) and the corresponding anticodons match the codon usage of mRNAs expressed in each state. Thus, a specific pool of tRNAs may be required to match the codon usage of genes important for oligodendrocyte differentiation and/or myelination or neuronal development or function, and reduced levels due to mutations in Pol III subunits or aaRS1 may contribute to the pathogenesis of HLD. Moreover, a recent study optimized next-generation sequencing of mature tRNAs to demonstrate a distinct expression profile of tRNA isodecoders in mouse CNS tissues compared to non-CNS tissues, with several isodecoders varying more than 4-fold, while total isoacceptor pools were relatively stable across these tissues (Pinkard et al., 2020). In an earlier study done by microarray, tRNA levels were found to vary across tissues, with the brain having among the highest levels of nuclear-encoded tRNAs (Dittmar et al., 2006). Together, these data suggest that CNS cell types could be particularly vulnerable to reduced tRNA levels, particularly for certain isodecoders that are more abundant in the CNS.

Consistent with this hypothesis, the Ackerman group identified the first instance of a tissue-specific mammalian tRNA gene, *n-Tr20*, which is exclusively expressed in the mouse CNS (Ishimura et al., 2014). *n-Tr20* encodes a tRNA-Arg-UCU isodecoder and contains a single nucleotide polymorphism (SNP) in the T stem loop in the C57BL/6J strain compared to other mouse strains. This results in accumulation of a precursor form of *n-Tr20* and decreased levels of the mature form and leads to increased ribosome pausing on AGA codons. On its own, the *n-Tr20* polymorphism was found to modulate seizure susceptibility and synaptic transmission (Kapur et al., 2020). Together with loss-of-function mutations in the recently characterized ribosome rescue factor genes *Gtpbp1* and *Gtpbp2*, the *n-Tr20* SNP leads to widespread neurodegeneration (Ishimura et al., 2014; Terrey et al., 2020), suggesting that these factors are essential to resolve ribosome pausing defects induced by decreased tRNA levels. Deletion of *n-Tr20* led to increased pausing at AGA codons genome-wide and reprogramming of the translatome and induced the integrated stress response (ISR) (Kapur et al., 2020), an important component of regulated translation (reviewed in Tahmasebi et al. (2018)). Moreover, a deletion in one of four expressed tRNA-Ile-UAU isodecoders (*n-Ti17*) decreased total tRNA-Ile-UAU levels and similarly increased the ISR in mouse brains, indicating that this is not specific to n*-Tr20* but rather a common response to deficient tRNA levels. Thus, specific tRNA isodecoders play essential roles in maintaining normal translation in mouse brains. Although isodecoders with CNS-specific expression have not yet been identified in humans, these results suggest that deficient expression of any single tRNA important for brain function could lead to translation deregulation. In the context of POLR3-HLD, reduced levels of specific isodecoder(s) important in certain spatio-temporal contexts could induce ribosome stalling at the corresponding codons and impair translation of proteins important for normal oligodendrocyte and/or neuronal function and underlie disease pathogenesis.

As described above, a subset of tRNAs were found to be downregulated in patient cells or in cell lines carrying POLR3-HLD mutations. The *POLR3A* Met852Val mutation (Figure 6), causing POLR3-HLD, significantly reduced pre-tRNA levels, but not those of selected mature tRNAs (Choquet et al., 2019a) in a cellular model of POLR3-HLD, whereas the *POLR3K* Arg41Trp mutation mildly decreased levels of mature tRNA^Met^ but not those of three other mature tRNAs (Dorboz et al., 2018). It should be noted that due to their extensive post-transcriptional modifications, tRNA expression levels are more difficult to determine by RT-qPCR or by RNA-sequencing, the primary methods used in these studies. DM-tRNA-seq (Zheng et al., 2015), ARM-seq (Cozen et al., 2015) and Hydro-tRNA-seq (Arimbasseri et al., 2015) were developed to overcome this obstacle and improved sequencing results. Recently, the mim-tRNA-seq (Behrens et al., 2021) and QuantM-tRNAseq approaches were published (Pinkard et al., 2020), which may help to further improve the quantification of mature tRNA expression levels, thereby allowing to determine whether POLR3-HLD can be the consequence of a modest reduction of pre-tRNA levels due to higher demand for translation in oligodendrocytes, or whether other mechanisms may also account for the development of this disease. Ribosome profiling in relevant cell types could also determine if ribosome stalling occurs at certain codons, as was observed with the *n-Tr20* polymorphism in mice.

Since HLDs are not only caused by mutations in genes encoding Pol III subunits, but also by alterations in several aaRS1 genes (e.g. *DARS1*; *EPRS1*; *RARS1*; Taft et al., 2013; Wolf et al., 2014a; Mendes et al., 2018, 2020), it has been speculated that changes in tRNA abundance (POLR3-HLD) or dysfunctions in the attachment of amino acids to tRNAs (HLD caused by aaRS1 mutations) could represent a unified disease-causing mechanism, in which reduced availability of specific aminoacylated tRNA(s) would lead to altered or insufficient translation by stalling ribosomes on the corresponding codons. A defect in aminoacylation was reported for disease-causing mutations in *KARS1, EPRS1* and *AARS1 in vitro* (Simons et al., 2015; Nakayama et al., 2017; Mendes et al., 2018; Itoh et al., 2019), while the aminoacylation activity of ArgRS1 was impaired upon some *RARS1* mutations but not with the most common mutation (Li G. et al., 2021). However, the potential impact of these mutations on translation was not investigated. Dominant mutations in several genes encoding tRNA aminoacyl synthetases are associated with Charcot-Marie-Tooth (CMT) disease and characterization of the corresponding mutants has demonstrated that aminoacylation activity is frequently not impaired. Instead, the mutations induce an alternative open conformation of the enzyme, which exposes a surface for new protein interactions (He et al., 2011; Blocquel et al., 2017; Bervoets et al., 2019; Blocquel et al., 2019; Sun et al., 2021), indicating a gain-of-function mechanism. In the case of leukodystrophies caused by bi-allelic mutations in genes encoding aaRS1, caused by hypomorphic mutations, further studies are required to determine if there is an underlying mechanism that involves translation deregulation and/or shares features with POLR3-HLD. Alternatively, aaRS1 possess numerous non-canonical functions (Wagasugi and Yokosawa, 2020; Yao and Fox, 2020) that could contribute to disease pathogenesis, although those differ between different aaRS1.

Upstream from their role in translation, misexpression of tRNA genes could affect their transcription, post-transcriptional processing and/or modifications. First, tRNAs are expressed from type 2 gene internal promoters, requiring TFIIIC and TFIIIB-β transcription factor complexes in order to recruit Pol III to the TSS (Dumay-Odelot et al., 2010; Figure 2B). High transcriptional efficiency at tRNA genes is at least in part enabled by facilitated recycling (Dieci et al., 2014). It is conceivable that Pol III mutations exert a negative effect on facilitated recycling, which could result in the decreased expression of tRNA genes observed in POLR3-HLD studies (Azmanov et al., 2016; Dorboz et al., 2018; Choquet et al., 2019a).

**FIGURE 7:**
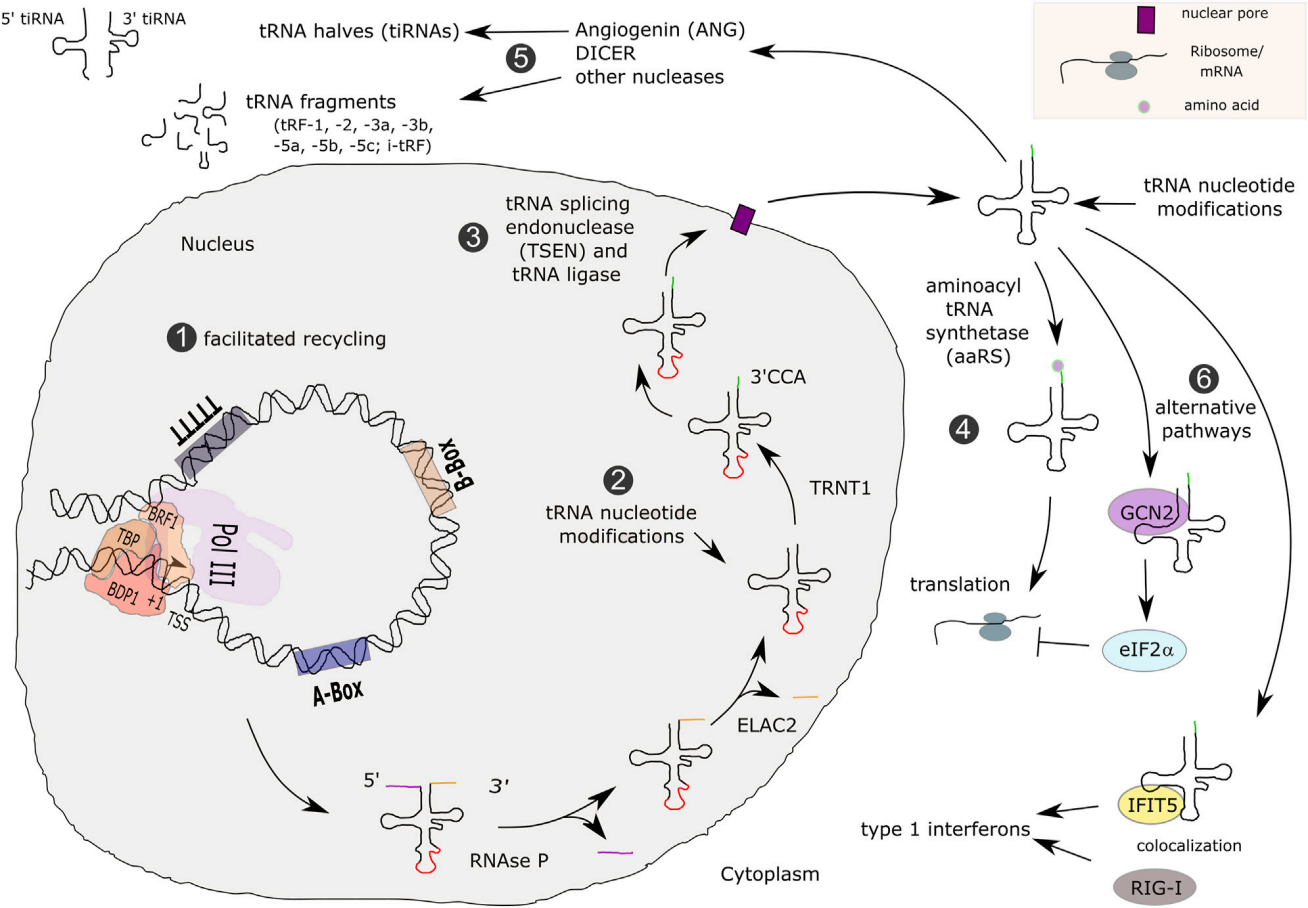
Transcription, maturation and functions of tRNAs. Type 2 promoter tRNA genes are transcribed by Pol III, possibly involving the facilitated recycling pathway, which allows transcription reinitiation in the absence of TFIIIC (1). Primary tRNAs contain 5′ leader and 3′ trailer sequences (colored lines) and a few human tRNAs contain introns (red line). tRNAs are processed by RNAse P, ELAC2 and TRNT1 (2), as well as spliced in the nucleus (3). Mature tRNAs are exported to the cytoplasm and loaded with amino acids for participating in translation (4). Other functions for tRNAs that were described involve enzymatic processing for yielding tRNA fragments (tRFs) or tRNA halves (tiRNAs) (5). In addition, they can enter alternative functional pathways (6).

Second, upon transcription termination, tRNAs undergo extensive post-transcriptional modifications including 1) the removal of the 5′ leader sequence by RNAse P (Jarrous, 2017), 2) processing of the 3′ end by ELAC2, the human orthologue of RNAse Z (Takaku et al., 2003; Siira et al., 2018), 3) the addition of CCA nucleotides to the 3’ terminus of tRNAs by the tRNA nucleotidyl transferase 1 (TRNT1) (Xiong and Steitz, 2006) and 4) removal of possible introns by the tRNA splicing endonuclease (TSEN) complex and CLP1 (Hayne et al., 2020). Subsequently, an average of 13 post-transcriptional modifications is brought upon individual tRNA molecules (reviewed in Pan (2018); tRNA transcription and maturation is summarized in Figure 7), many of which are important for normal brain function (reviewed in Ramos and Fu (2019)). Post-transcriptional modifications alter local and overall tRNA folding, affecting their stability (reviewed in Ramos and Fu (2019)). The half-life of precursor (pre)-tRNAs was estimated to be 15 to 30 min whereas it is about 100 h for mature tRNAs (Choe and Taylor, 1972). Most of these processing steps have been associated with neurological disorders: changes in modifications of nucleotides in the anticodon stem loop or at transitions from stem to D-loop or T-loop structures were shown to be related to the development of neurodevelopmental disorders, including intellectual disabilities or amyotrophic lateral sclerosis (Freude et al., 2004; Bento-Abreu et al., 2018; Sharkia et al., 2019). Several enzymes involved in pre-tRNA processing and tRNA post-transcriptional modification are associated with inherited neurodegenerative disorders (reviewed in Schaffer et al. (2019)). Thus, the CNS appears to be particularly vulnerable to any defect in tRNA metabolism, further indicating that reduced tRNA expression in POLR3-HLD is a likely mechanism underlying dysfunction of neurons and/or oligodendrocytes.

Of particular relevance to POLR3-HLD, accumulation of a tRNA processing intermediate retaining the 5’ leader was observed in a *S. cerevisiae* mutant strain carrying two POLR3-HLD mutations at the homologous positions in *Rpc160* (yeast homolog of *POLR3A*) (Moir et al., 2021). Although this was not sufficient to impair mature tRNA levels (Moir et al., 2021), it indicates that tRNA processing could also be affected by POLR3-HLD, which may have particular importance in certain tissues. In *S. pombe*, introduction of two homologous POLR3-HLD mutations led to decreased transcription of three tRNA genes, but also to increased tRNA N2,N2-dimethyl G26 (m^2^_2_G26) modification efficiency (Arimbasseri et al., 2015). Global repression of Pol III transcription through rapamycin treatment yielded a similar effect, both in *S. pombe* and in human HEK293 cells, suggesting that this response is conserved. Thus, reduced transcription of tRNA genes may lead to increased modification efficiency of tRNAs, which could be detrimental for brain function. Furthermore, stress-correlated modification of tRNAs was reported to occur (Gu et al., 2014), which might be affected by lower tRNA transcription rates and subsequently affect aminoacylation and translation.

Aspects of mRNA and tRNA modification have also been shown to be coordinated (Ontiveros et al., 2020; Levi and Arava, 2021). For example, the enzyme TRMT10A, which is known for depositing m^1^G on tRNAs, also influences m^6^A deposition on mRNAs by interacting with FTO (Ontiveros et al., 2020). Depletion of TRMT10A decreased m^1^G levels on tRNAs but increased m^6^A levels on mRNA. Some pseudouridine synthases were also shown to modify both tRNAs and mRNAs (Borchardt et al., 2020). This opens the door to the possibility that alterations in transcription of tRNAs in POLR3-HLD could influence post-transcriptional modification of mRNAs, with reduced levels of tRNAs liberating more enzymes for acting on mRNAs, thus modulating processing, stability and/or translation of these mRNAs.

Finally, tRNAs have non-canonical functions outside of translation that could be misregulated in POLR3-HLD. tRNAs were shown to bind to and modify the activities of proteins that are not directly involved in translation control. For instance, it was demonstrated that tRNA-GCN2 kinase interactions regulated the phosphorylation of eIF2α, thereby reprogramming translation towards general repression whilst activating translation of selected mRNAs (Dong et al., 2000; Castilho et al., 2014; Figure 7). In addition, the interferon-induced tetratricopeptide repeat 5 (IFIT5) protein binds tRNAs, thereby modulating double-stranded DNA sensing receptor RIG-I and being involved in regulating type I interferon response (Katibah et al., 2013; Figure 7). Such mechanisms may also be sensitive to changes in tRNA transcription and could therefore be of importance for the development of POLR3-HLD.

### 7SL RNA – Transcription, Structure and Functions

7SL RNA is a major component of the signal recognition particle (SRP). In addition to 7SL RNA, the SRP is composed of the SRP9, 14, 54, 68 and 72 proteins. It is responsible for co-translational targeting of nascent secretory and transmembrane peptides to the endoplasmic reticulum (ER) through interaction with its SRP receptor (Lakkaraju et al., 2008; Akopian et al., 2013). Pol III transcription of the 7SL gene is directed by promoter elements that are located within the transcribed region (A- and B-boxes), as well as by TATA-like, ATF/CRE and STAF-binding sequences upstream of the TSS (Ullu and Weiner, 1985; Bredow et al., 1990a; Englert et al., 2004; Dumay-Odelot et al., 2014) (hybrid promoter; Figure 2D).

Genomic occupancy of POLR3G (RPC32α; Haurie et al., 2010), BRF1/TFIIIB-β (Teichmann and Seifart, 1995; Wang and Roeder, 1995; Mital et al., 1996), GTF3C4/TFIIIC63 (Hsieh et al., 1999) and BDP1 (Schramm et al., 2000; Teichmann et al., 2000) was analyzed, suggesting that transcription of the 7SL gene is carried out *in vivo* by TFIIIC, TFIIIB-β and Pol III (Canella et al., 2010; Oler et al., 2010). *In vitro*, 7SL transcription was shown to be stimulated by ATF (Bredow et al., 1990b). *Ex vivo*, 7SL was identified as the most abundant non-rRNA transcript in two cell lines (Boivin et al., 2018).

**FIGURE 8:**
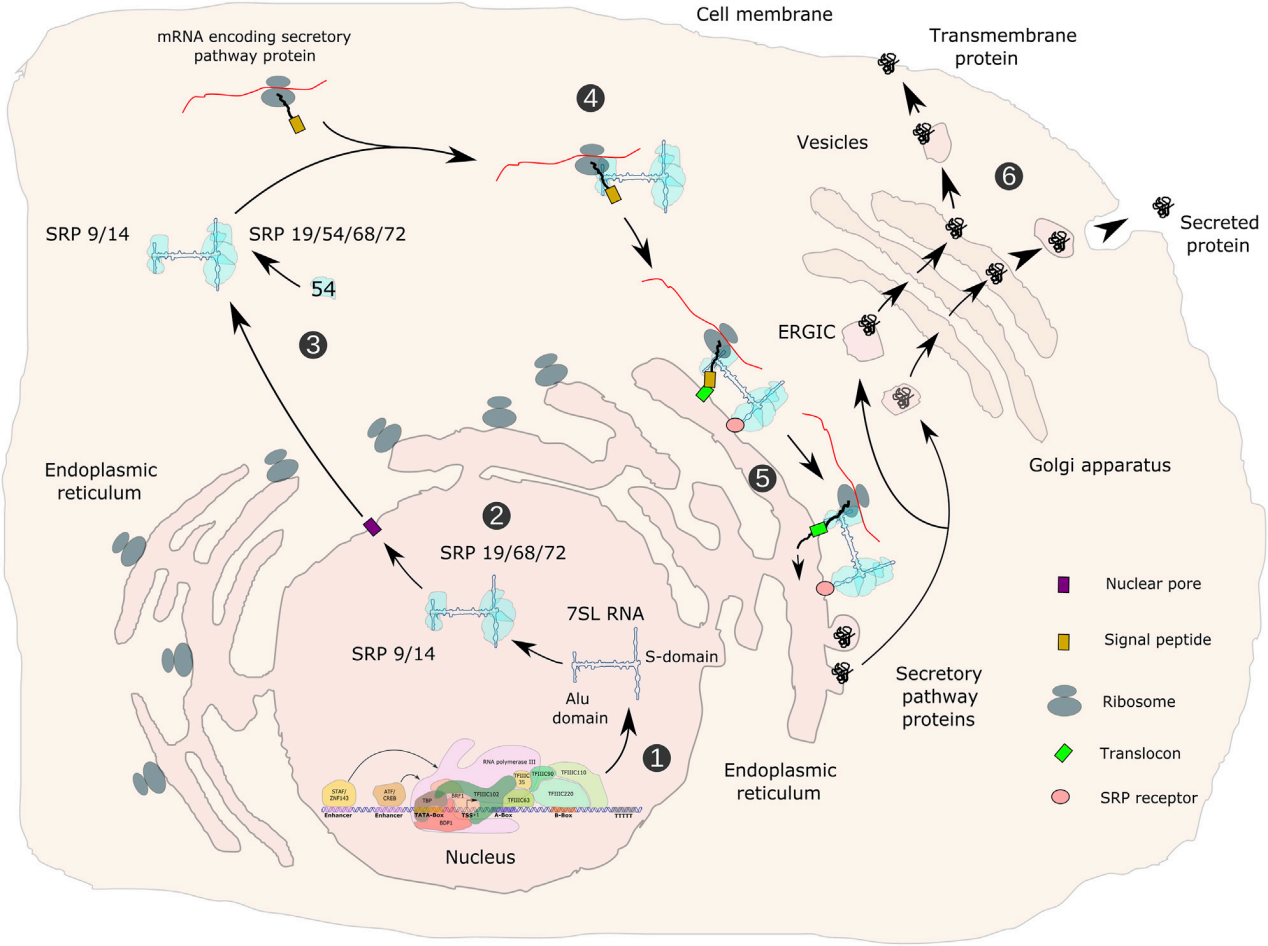
Transcription of the 7SL RNA gene, assembly of the signal recognition particle and functions in the secretory pathway. 7SL RNA gene transcription is directed by gene-internal A- and B-boxes as well as by an ATF/CREB-response element and a distal sequence element (DSE) upstream of the TSS. Transcription is initiated by TFIIIC- and TFIIIB-β-dependent recruitment of Pol III (1). The SRP 9/14/19/68/72 pre-signal recognition particle (pre-SRP) is assembled in the nucleus (2), exported to the cytoplasm and completed with SRP54 (3). SRP recognizes hydrophobic signal peptides in nascent polypeptide chains of secretory pathway proteins (4), arrests translation, translocates arrested ribosomes to the endoplasmic reticulum (ER), where translation resumes and proteins are translocated either into the lumen or into the membrane of the ER (5). Proteins of the secretory pathway are either incorporated into membranes or exported from cells (6). ERGIC refers to the ER-Golgi-intermediate compartment.

The 300 nt human 7SL RNA contains two domains that were identified by micrococcal nuclease digestion. Base pairing of the 5′ and 3’ parts of 7SL RNA forms the Alu domain, whereas the central part folds into the S domain (Gundelfinger et al., 1983; Zwieb, 1985; Figure 8). The Alu domain represents the binding site for SRP14/SRP9, whilst the S-domain is recognized by SRP19, SRP54 and the SRP68/SRP72 heterodimer, altogether composing the SRP (Gundelfinger et al., 1983). A pre-SRP, consisting of 7SL RNA and SRP proteins 9, 14, 19, 68 and 72 is assembled in the nucleus. Upon export to the cytoplasm, it is completed by the addition of SRP54 (Massenet, 2019; Figure 8). SRP54 within the S-domain-associated protein complex recognizes a N-terminal hydrophobic signal sequence in nascent peptide chains of proteins. The SRP14/SRP9-containing Alu domain in turn interacts with translation elongation factor binding sites within cytoplasmic ribosomes, thereby inducing an elongation arrest (Halic et al., 2004; Voorhees and Hegde, 2015). Proteins containing a signal peptide are either secreted or are an integral part of the cell membrane. Stalled ribosomes are then targeted to the Sec61 core component of the translocon within the ER via a GTP- and SRP54-dependent process, resulting in proteins being synthesized and translocated either into the lumen or into the membrane of the ER and secreted or delivered to the cellular membrane (Figure 8). GTP hydrolysis triggers the release of SRP from the Sec61 translocon, allowing translation to resume (Fulga et al., 2001; Pool, 2005). Depletion of SRP14, SRP54 or SRP72 in HEK293 or HeLa cells leads to decreased 7SL RNA levels, inefficient ER targeting and impaired post-ER membrane trafficking (Lakkaraju et al., 2007). Thus, decreased 7SL RNA levels in POLR3-HLD may impair translocation of secreted proteins to the ER, which could contribute to the pathophysiology of POLR3-related disorders in several different cell types. 7SL RNA is at its highest level of expression in the hypothalamus compared to other non-CNS tissues (Castle et al., 2010). The expression of 7SL RNA was demonstrated to be positively regulated during differentiation of mouse embryonic stem cells into a differentiated heterogeneous population of neurons and glial cells (Skreka et al., 2012), suggesting that it may be of particular importance in these cell types, both of which are affected in POLR3-related disorders.

In oligodendrocytes, proteolipid protein (PLP) is a major myelin protein that is targeted to the ER and follows the secretory pathway to reach the site of myelination (Woodward, 2008). Reduced SRP function could potentially impair PLP trafficking and contribute to POLR3-HLD pathogenesis (Figure 4). In depth analysis of such mechanism may require the establishment of appropriate experimental systems, including the study of primary oligodendrocytes derived from iPSCs and/or in co-culture with other CNS cell types. For example, iPSCs from individuals carrying mutations in the gene encoding PLP, which cause Pelizaeus-Merzbacher HLD, were differentiated into oligodendrocytes to show mislocalization of PLP to the ER and to identify modulators of ER stress (Numasawa-Kuroiwa et al., 2014; Nevin et al., 2017). Similar experiments could be undertaken with iPSCs from POLR3-HLD patients to determine if PLP or other myelin proteins are mislocalized. In addition, three-dimensional growth of human iPSC-derived oligodendrocytes in organoid cultures (Marton et al., 2019) may allow reproducing a cellular environment that better reflects the *in vivo* situation. Cells grown under these conditions may show higher dependency on optimal Pol III transcription and may therefore be more vulnerable to protein mislocalization in the case of reduced 7SL RNA expression.

In neurons, protein trafficking through the ER is crucial for both dendritic and axonal function, including for synaptic plasticity and neurotransmitter trafficking (reviewed in Ramirez and Couve (2011), Kennedy and Hanus (2019)). In mouse motor neurons, 7SL RNA was found to be more abundant in axons than in the somatodendritic compartment, indicating an important role in axons (Briese et al., 2016). Moreover, the importance of ER function in neurons is exemplified by the fact that around half of hereditary spastic paraplegias (HSPs) are caused by mutations in genes encoding ER-shaping proteins (Ozturk et al., 2020). At least one HSP-associated mutation causes a kinetic delay in ER protein secretion (Slosarek et al., 2018). Spasticity is observed in patients with POLR3-related spastic ataxia, suggesting that pyramidal neurons, the primary affected cell type in HSPs, are also involved in POLR3-related disorders. A possible hypothesis is that reduced 7SL RNA levels could affect ER targeting in these neurons or others, leading to the observed neurodegeneration.

Another connection between 7SL RNA and neurodegeneration is the fact that *in vivo* assembly of the SRP complex depends on the survival of motoneuron (SMN) complex (Piazzon et al., 2013; Massenet, 2019), which is responsible for assembly of ribonucleoprotein (RNP) complexes, most notably the spliceosomal snRNPs (Li et al., 2014), and is composed by the SMN protein and Gemin2-8 proteins. Mutations in *SMN1*, encoding the SMN protein, cause spinal muscular atrophy (SMA) (Lefebvre et al., 1995; Wirth, 2000; Wirth et al., 2020). Reduced levels of 7SL RNA were detected in the spinal cord but not in the brain and heart of an SMA mouse model (Piazzon et al., 2013), suggesting that 7SL RNA levels are regulated by SMN function in a cell-type specific manner. Recently, loss-of-function mutations in *GEMIN5* were found to cause a neurodevelopmental syndrome that includes cerebellar ataxia (Kour et al., 2021). *GEMIN5* mutations decreased levels of snRNP complexes *in vivo* and disrupted SMN complex assembly *in vitro*. Thus, dysfunction of the SMN complex can specifically affect cells of the CNS. Since 7SL RNA was found to compete with U1 and U2 snRNPs for binding to SMN complexes *in vitro* (Piazzon et al., 2013), it is possible that similar competition occurs *in vivo* when SMN function is compromised, resulting in impaired protein secretion that could contribute to the disease phenotypes. Future investigation of protein secretion in SMA and other SMN-related disorders will help clarify the potential role of 7SL RNA in neuronal dysfunction.

Reduced 7SL RNA levels may also contribute to other disease phenotypes, such as the digestive dysfunction observed in patients with *POLR3K* mutations (Dorboz et al., 2018) and in *polr3b* mutant zebrafish (Yee et al., 2007). Indeed, intestinal epithelial cells are highly secretory and are sensitive to ER stress and unfolded protein response (reviewed in Coleman and Haller 2019). Future experiments are required to determine the role of the POLR3K-POLR3B interface and of 7SL RNA in intestinal cell homeostasis.

### BC200 - Transcription, Structure and Functions

BC200 (BCYRN1 – brain cytoplasmic RNA 1) is a monomeric Alu RNA that is predominantly expressed in the brain of primates. It was discovered by northern blot analyses (Watson and Sutcliffe, 1987) employing an identifier (ID) sequence as a probe. This ID element, the rodent-specific BC1 RNA of 154 nt, was previously shown to be specifically expressed in rat brain (Sutcliffe et al., 1982, 1984). BC1 RNA is derived from a tRNA^Ala^ retrotransposition event (Daniels and Deininger, 1985), whereas the 200 nt primate BC200 RNA is a 7SL RNA-derived exapted monomeric Alu element (Watson and Sutcliffe, 1987; Brosius, 1999). As a consequence, BC1 and BC200 RNA share little sequence homology and are thought to be functional analogs rather than true homologs. The fact that two independent retrotransposition events resulted in the generation of distinct but related brain-specific RNAs indicated that these RNAs fulfil important roles within the brain. However, KO of the BC1 gene in mice resulted in healthy animals which showed at the first sight only discrete neurological abnormalities such as neuronal hyperexcitability (Skryabin et al., 2003; Zhong et al., 2009). Closer inspection in later behavioral trials demonstrated that BC1 KO mice had impaired cognitive abilities (Chung et al., 2017; Iacoangeli et al., 2017). In addition to the neuron-specific expression in healthy animals, BC1 and BC200 expression was also detected in tumor samples and tumor cell lines (for review see Samson et al. (2018)). A recent study using qRT-PCR found that BC200 RNA levels were comparable in primary cell lines and tumor cell lines from the same tissue. Expression of BC200 RNA in three primary or non-tumorigenic cell lines was also surprisingly similar to GAPDH mRNA levels (Booy et al., 2017).

The BC200 gene contains classical type 2 intragenic promoter elements (A-box and B-box) and in addition a TATA-like sequence upstream of the TSS (hybrid promoter). Sequences up to 100 nucleotides upstream of the TSS were suggested to be important for BC200 transcription efficiency in transient transfection experiments. Stepwise deletion of these sequences led to a gradual decline in transcription rate without changing the ability of Pol III to correctly recognize the TSS. In addition, mutation of gene internal A- or B-boxes abolished transcription of the BC200 gene (Kim et al., 2017). These results indicate that gene internal promoter elements are the crucial determinants of BC200 expression and TSS selection. The importance of gene internal control elements and of sequences upstream of the TSS were also demonstrated by *in vitro* transcription of rodent BC1 gene (Martignetti and Brosius, 1995). In addition, BRF1, TFIIIC and Pol III subunits, but not BRF2 were detected by ChIP-seq at the BC1 gene promoter in mice, underscoring that it is regulated by gene internal promoter elements and stimulated by regulatory elements upstream of the TSS (Carrière et al., 2012).

As the only Pol III transcript with brain-specific expression, BC200 RNA represents an attractive candidate for a role in POLR3-related disorders. In the tumor cell line MO3.13, which has characteristics of oligodendrocyte progenitor cells (OPC) (McLaurin et al., 1995), KO of BC200 led to significant gene expression changes (Choquet et al., 2019a), suggesting a function for BC200 RNA in OPCs, although these findings must be confirmed in primary cells to draw definite conclusions. It should be noted that early *in situ* hybridization experiments did not detect BC200 RNA expression in adult brain white matter (Tiedge et al., 1993), but this does not exclude the possibility that BC200 RNA is expressed in OPCs or in oligodendrocytes earlier in development, such as when myelination occurs, especially given that recent studies have detected BC200 RNA expression in non-neuronal primary cell lines (Booy et al., 2017; Choquet et al., 2019a), albeit at lower levels than in the brain. Additional functional studies will be required to determine if BC200 RNA is important for other cell types, and expression profiling in different CNS cell types from fetal and adult tissues will help establish how BC200 RNA is modulated spatially and temporally.

Alternatively or in addition, impaired expression of BC200 RNA may contribute to some of the neuronal phenotypes (e.g. cerebellar, striatal) observed in POLR3-related disorders. Functional studies on BC200 RNA have mostly been performed *in vitro*, in tumor cell lines or by analogy with BC1 RNA. Nonetheless, the many identified interacting partners and potential functions for this non-coding RNA provide hypotheses as to how it may contribute to the pathogenesis of POLR3-related disorders.

**FIGURE 9:**
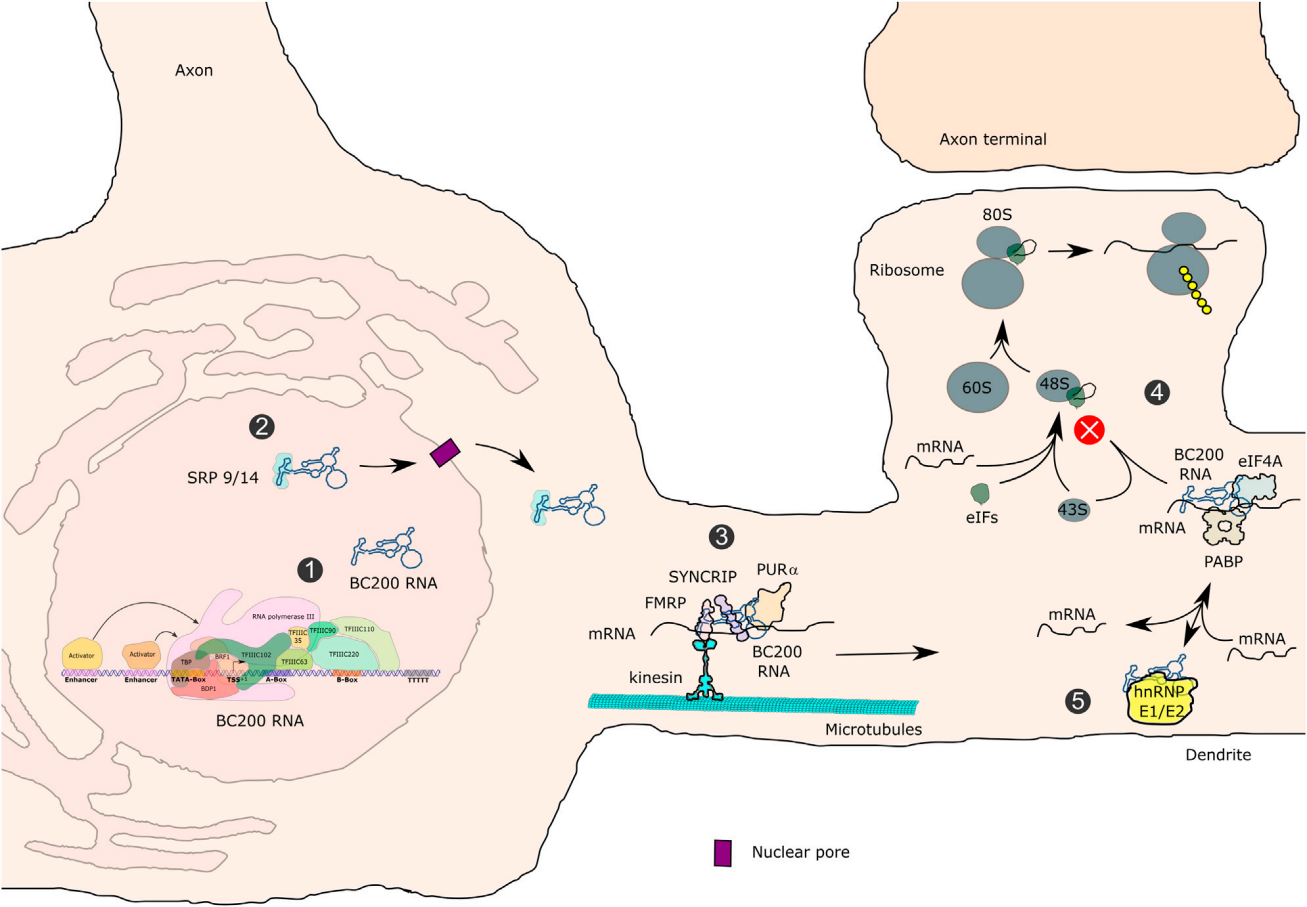
Transcription of the BC200 RNA and its functions in mRNA transport and regulation of translation. BC200 RNA gene transcription depends on TFIIIC bound to gene-internal A- and B-boxes, which allows the subsequent recruitment of TFIIIB-β and Pol III. Sequences upstream of the TSS including a TATA-like box at -30 regulate transcription activity (1). BC200 RNA assembles with SRP 9/14 in the nucleus (2) and is exported to the cytoplasm where it was shown to interact with a variety of proteins. BC200 was shown to interact with FMRP, SYNCRIP and Pur-alpha, suggesting a role in mRNA transport similar to what was demonstrated for BC1 in complex with these proteins (3). In association with eukaryotic initiation factor 4A (eIF4A) and poly A binding protein (PABP), BC200 RNA prevents the assembly of the 48S ribosomal subunit (4). This repression of translation can be counteracted by hnRNP E1/E2 binding to BC200 RNA *in vitro* (5).

According to a structural model of BC200 RNA, the first 120 nucleotides at the 5′-end, together with nucleotides 175–200 of the C-terminal unique region, fold into an Alu-domain. The 5′ part of the Alu-domain and the unique C-rich domain at the 3′-end of BC200 RNA are separated by a loop-forming A-rich domain. The Alu-domain of BC200 is highly similar to that of 7SL RNA (Sosińska-Zawierucha et al., 2018; Figure 9). Consequently, the 7SL-interacting SRP9/14 heterodimer was also shown to interact with BC200 RNA (Bovia et al., 1997; Kremerskothen et al., 1998) and possible consequences on translation inhibition were discussed. Other proteins interacting with BC1 and/or BC200 RNAs were described, including Pur α (Kobayashi et al., 2000; Johnson et al., 2006), Fragile X Mental Retardation Protein (FMRP; Zalfa et al., 2003), Poly(A)-binding Protein (PABP; Muddashetty et al., 2002), Synaptotagmin-binding cytoplasmic RNA interacting Protein (SYNCRIP/hnRNP Q1; Duning et al., 2008), RNA helicase associated with AU-rich element (RHAU/DHX36; Booy et al., 2016), eukaryotic translation initiation factor 4A (eIF4A; Lin et al., 2008) and heterogeneous nuclear ribonucleoproteins E1 and E2 (hnRNP E1 and E2; Jang et al., 2017).

These proteins were proposed to influence the stability and/or the export of BC200 from the nucleus to the cytoplasm (SRP9/14), mRNA transport in neuronal dendrites (Pur α, FMRP, SYNCRIP) and/or interfere with BC1/BC200 effects on translation. Whilst eIF4A and PABP are targets of translational inhibition by BC200/BC1 RNAs (Lin et al., 2008), hnRNP E1 and E2 were proposed to counteract BC200-mediated translation inhibition (Jang et al., 2017; Figure 9). The helicase RHAU/DHX36 was shown to mediate the binding of unwound G-quadruplexes to BC200 (Booy et al., 2016), thereby possibly indirectly intervening with translation. The question of whether BC1/BC200 stimulates or inhibits translation has not been definitively solved. Most reports indicate that these RNAs contribute to repression of translation in postsynaptic dendrites through interactions with eukaryotic initiation factors (eIFs) 4A and 4B (reviewed in Iacoangeli and Tiedge (2013)), with most of these experiments performed *in vitro* or only for BC1 RNA.

Recent studies performed in human cell lines indicate that the role of BC200 RNA goes beyond what was learned from *in vitro* experiments or by analogy with BC1 RNA. Indeed, a recent report shows that depletion of BC200 in MCF-7 breast cancer cells resulted in the reduction of translation (Booy et al., 2020). Thus, the impact of BC1/BC200 RNAs on translation may be context-dependent and vary in neurons where these RNAs act as translational repressors inhibiting eIF4A helicase activity and in tumor cells where translation is executed at sites within the cytoplasm that differ largely from specialized compartments such as synapses. Moreover, the BC200 interactome was analyzed in three transformed cell lines (MCF-7; MDA-MB231; HEK293T) by exogenous expression of a 3′-end labeled BC200 RNA (Booy et al., 2018). This confirmed previous interactors of BC200 RNA (e.g. SRP9/14, PABPC1, DDX36) but also identified new interactors (e.g. TRIM24, HNRNPK, CSDE1), several of which are involved in regulating RNA stability. Some binding partners may influence the stability of BC200 RNA itself, while others may be functional partners. A reciprocal interaction was shown for at least one binding partner, in which CSDE1 regulates BC200 RNA levels, while BC200 RNA influences CSDE1 post-transcriptional regulation, likely by affecting translation rate or protein stability. In addition, BC200 RNA was also found to regulate alternative splicing (Singh et al., 2016) and mRNA stability (Shin et al., 2017a; Shin et al., 2017b) of specific transcripts in cell lines, suggesting that its role does go beyond the analogies drawn from studying BC1 RNA and that BC200 RNA may modulate RNA processing or stability of the same or other target mRNAs in CNS cells. Establishing whether BC200 RNA accomplishes these functions or interacts with the same protein partners in normal cells such as neurons in addition to tumor cells represents an important avenue of future research. Furthermore, characterizing the mRNA interactome of BC200 RNA in normal and tumor cells from different tissues may help to clarify its function(s).

Interestingly, repression of myelin basic protein (MBP) translation during transport is mediated in part by hnRNP-E1 (Torvund-Jensen et al., 2014), which was also shown to regulate BC200 RNA function *in vitro* (Jang et al., 2017), providing a tenuous but potential link between BC200 RNA and myelination.

The data summarized here indicate that BC200 RNA plays important roles in post-transcriptional mRNA regulation, with most studies so far having focused on its function in postsynaptic translation. The Alu domain in BC200 RNA associated with SRP9/14 proteins resembles the translation arrest domain of the 7SL RNA-containing SRP, suggesting that it is likewise involved in translation inhibition. As a consequence, reduced BC200 RNA levels may lead to imbalanced postsynaptic translation. Although oligodendrocytes are the primary cell type affected in POLR3-HLD, a direct neuronal dysfunction is thought to be responsible for the neuronal loss observed in POLR3-HLD (e.g. cerebellum) and other POLR3-related disorders (e.g. cerebellum, striatum) (Wolf et al., 2014a; Azmanov et al., 2016; La Piana et al., 2016; Minnerop et al., 2017; Perrier et al., 2020a). The role of BC200 RNA in dendrites could affect the function and integrity of neurons in these brain regions. Furthermore, as mentioned above, a potential role for BC200 RNA in oligodendrocytes and/or their progenitor cells could contribute to the hypomyelination phenotype.

## Linking Mutations in Genes Encoding Subunits of Pol III to the Innate Immune System

**FIGURE 10:**
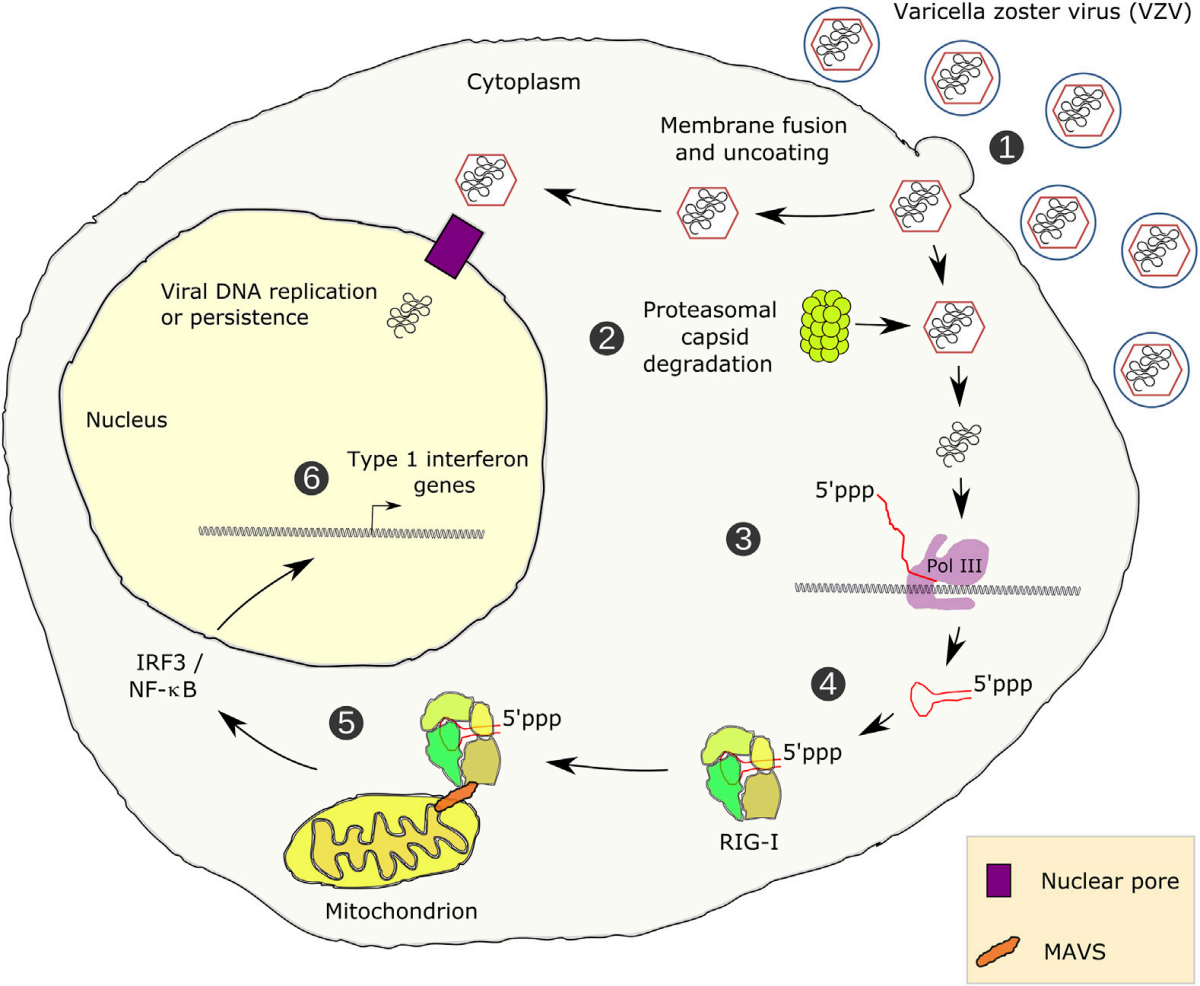
Cytoplasmic transcription by RNA polymerase III in innate immune defense. Upon infection (1), the varicella zoster virus (VZV) DNA is found in the nucleus and the cytoplasm (Mahalingam et al., 1999; Zerboni et al., 2014). Although not specifically reported for VZV, cytoplasmic liberation of DNA from capsids may involve proteasomal degradation (2), as was described for herpes simplex virus 1 (HSV-1; Horan et al., 2013). Cytoplasmic AT-rich VZV DNA is transcribed by Pol III (3), the 5′ tri-phosphorylated RNA folds into stem-loop structures, interacts with the retinoic acid-inducible gene I (RIG-I) (4), which, via mitochondrial antiviral signaling (MAVS) protein and NF-kB/IRF3 transcriptional activators (5) increases transcription of type I interferon genes (6).

Although Pol III is best known for the nuclear transcription of small non-coding RNA genes, its function in the immune response is becoming increasingly clear. The discovery that Pol III does not only participate in nuclear transcription of small RNAs, including viral RNAs, but is also involved in the detection of invading DNA, expanded cellular activities of this enzyme to innate immunity. Pol III recognizes double-stranded transfected linear (ds)AT-rich DNA in the cytoplasm and transcribes it into 5’ triphosphorylated RNA, which triggers the activation of retinoic acid-inducible gene I (RIG-I). RIG-I activation is dependent on the AT-content of the produced RNA since it can be abolished by insertion of GC sequences (Chiu et al., 2009). Activated RIG-I signals to the mitochondrial antiviral signaling protein (MAVS), resulting in the induction of the production of type 1 interferons (Ablasser et al., 2009; Chiu et al., 2009; Figure 10). The Pol III-dependent pathway of inducing innate immune response by production of RNAs from AT-rich DNA is complementary to the Cyclic GMP–AMP synthase (cGAS) pathway, which is activated by binding to DNA from invading microbes and production of cGAMP (Sun et al., 2013; Luecke et al., 2017; reviewed in; Tan et al. (2018)). Cytoplasmic DNA recognized by Pol III is derived from infections with Gram-negative bacteria (*Shigella flexneri* or *Legionella pneumophilia*), Gram-positive bacteria (*Listeria* monocytogenes) or from viral infection (herpes simplex virus 1), suggesting that both bacterial and viral sources can trigger the Pol III-dependent innate immune system (Chiu et al., 2009; Pollpeter et al., 2011; Jehl et al., 2012; Crill et al., 2015). In addition to defending cells against acute infectious threats, Pol III nuclear transcription also contributes to induction of interferon production in cells having been transfected with adenoviral DNA (Minamitani et al., 2011) or latently infected by the Epstein-Barr virus. Thus, nuclear Pol III transcribing adenoviral VA RNAs or Epstein-Barr viral EBER1 and EBER2 genes is also able to trigger RIG-I-dependent type 1 interferon production (Samanta et al., 2006; Minamitani et al., 2011).

**FIGURE 11:**
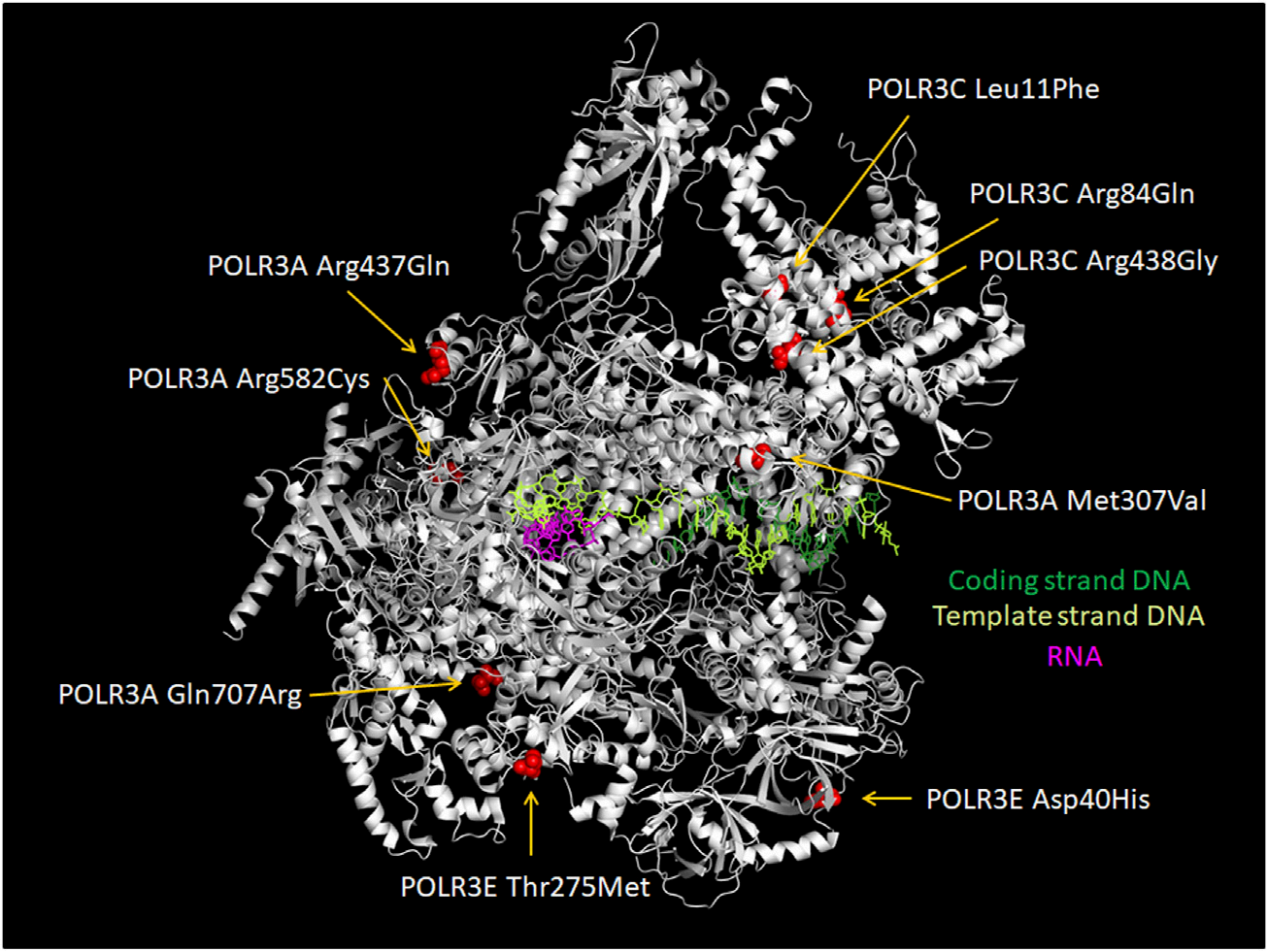
Distribution of mutations in RNA polymerase III subunits associated with impaired innate immune defense. Spatial distribution of amino acid mutations that are associated with severe immune deficiency (POLR3E Asp40His) or with varicella zoster virus (VZV) encephalitis and/or pneumonitis (other displayed mutations). Mutated amino acids are depicted as spheres and highlighted in red. Individual mutations in subunits POLR3A, 3C, 3E are appropriately designated. The mutation POLR3F Arg50Trp cannot be displayed since the corresponding sequence was not resolved in the cryo-EM structure (PDB:7ae1). The Figure was created using Pymol.

Consistent with this role of Pol III in innate immunity, rare heterozygous genetic variants in the genes encoding Pol III subunits POLR3A (RPC1), POLR3C (RPC3), POLR3F (RPC6) and POLR3E (RPC5) were shown to strongly impair immune response to varicella-zoster virus (VZV) infections in humans. This reduced immune response resulted in the development of VZV pneumonitis (mutations in *POLR3A*, *POLR3C*) or of VZV encephalitis (mutations in *POLR3A*, *POLR3C*, *POLR3E* and *POLR3F*) (Ogunjimi et al., 2017; Carter-Timofte et al., 2018a, 2019; Figure 11). Importantly, Pol III mutations associated with susceptibility towards VZV infections have not been linked to POLR3-HLD or other neurodegenerative diseases (Ogunjimi et al., 2017). Together with the different mode of inheritance and the fact that these patients are healthy until VZV infections, this indicates that pathogenic mechanisms are likely fundamentally different.

Heterozygous missense variants affecting VZV immune response were first described in *POLR3A* and *POLR3C*. These variants led to reduced interferon production in peripheral blood mononuclear cells (PBMCs) from patients, which could be rescued *in vitro* by introduction of wild type alleles of the mutated genes into these cells, suggesting defects in Pol III cytoplasmic function. Nuclear 5S rRNA gene transcription was not affected in PBMCs carrying *POLR3A*- and *POLR3C* mutations (Ogunjimi et al., 2017). Although it remains possible that expression of other nuclear Pol III transcripts is impaired in these cells, these data suggest that cytoplasmic DNA transcription by Pol III has unique requirements in terms of polymerase-DNA- or polymerase-associated protein-interactions compared to the nuclear gene expression by the Pol III enzyme. In line with this hypothesis, structural studies of human Pol III found that mutations associated with severe VZV infections mapped to the periphery/surface of Pol III (Ramsay et al., 2020; Girbig et al., 2021) and made few contacts with other residues or subunits (Type IV in Girbig et al. (2021)), in contrast to mutations associated with recessive POLR3-related disorders. One possibility, which is at present favored, would be that cytoplasmic AT-rich DNA does not require the presence of Pol III promoter elements (A-, B- or TATA-boxes) and transcription is therefore independent of Pol III transcription factors. However, it cannot be excluded that TATA-like elements, which may be present in AT-rich DNA and may result in the recruitment of TATA-binding protein (TBP)-containing TFIIIB-α or -β transcription initiation factors, would in turn enable the recruitment of Pol III to participate in cytoplasmic immune response. Consistent with this hypothesis, it should be noted that all Pol III transcription factors have been detected in the cytoplasm and would therefore be available for transcription of cytoplasmic DNA (for example, TBP: Hardivillé et al., 2020; BRF1: Mital et al., 1996; BDP1: Weser et al., 2004; TFIIIC: Dumay-Odelot et al., 2007; Schneider et al., 1989; Pol III: Jones et al., 2000; Haurie et al., 2010). Furthermore, it should be considered that viral genomes are not present in the cytoplasm as short dsDNA with possibly 3′ overhanging ssDNA elements comparable to AT-rich DNA fragments, but may be present as hundreds of kilobases long DNA elements without structural elements that would be required for factor-independent Pol III transcription initiation. This means that these viral DNAs cannot be transcribed highly efficiently like the short AT-rich dsDNAs without Pol III transcription factors but may have to rely on transcription factor-dependent mechanisms to elicit an efficient RIG-I-dependent immune response. Furthermore, it should be considered that the VZV genome in human cells is circular (Cohen, 2010) and thus lacks free 3′ overhangs which would be indispensable for factor-independent Pol III transcription initiation. As a consequence, it seems quite possible that the cellular, Pol III transcription-dependent immune response could also rely on Pol III transcription factors in the cytoplasm. Future research will clarify whether RIG-I activation by Pol III-transcribed RNAs occurs independently from transcription factor or with the involvement of TFIIIB and TFIIIC. Regardless of whether Pol III transcription factors are involved in these processes or not, the fact remains that sequences transcribed by Pol III must be AT-rich to elicit a RIG-I response.

Mechanistically, an observation may also link cytoplasmic immune response to a helicase activity intrinsic to Pol III. Arginine 84 in POLR3C (RPC3) was shown to be replaced by glutamine (Arg84Gln) in a patient who developed VZV-induced encephalitis (Ogunjimi et al., 2017; Figure 11). Interestingly, a mutation of the same residue of POLR3C, Arg84Ala, was shown to result in a defect in its intrinsic helicase activity *in vitro* (Ayoubi et al., 2019). This finding could be in line with a model in which Pol III helicase activity is required for dsDNA unwinding of viral cytoplasmic DNA.

Interestingly, a rare homozygous variant in *POLR3E* (Asp40His) was recently identified in a child with recurrent and systemic viral infections and Langerhans cell histiocytosis, indicating a more severe immune deficiency than in individuals carrying heterozygous mutations conferring specific VZV susceptibility who are otherwise healthy. Induction of interferon expression was triggered by the CG-rich (57,5%; Marti-Carreras and Maes, 2019) genome of human cytomegaly virus (HCMV) in control cells and abolished in patient cells homozygous for the POLR3E Asp40His mutation (Ramanathan et al., 2020), suggesting a different mechanism than the reported Pol III immune response to AT-rich DNA. The authors found that HCMV and sindbis virus infection induced POLR3E expression in control cells. Transfection of plasmid DNA also induced POLR3E expression and led to increased expression of 5S rRNA and a tRNA gene. In contrast, ectopic expression of wild-type POLR3E had a lower effect on expression of these Pol III target genes compared to the empty vector, and this response was absent or much lower with ectopic expression of mutant POLR3E. Finally, expression of the mutant POLR3E impaired formation of Pol III initiation complexes. These results suggest a role for Pol III nuclear transcription in the response to foreign viral and non-viral nucleic acids. Although the identification of additional patients with a similar phenotype is necessary to confirm that the POLR3E mutation is indeed causal, these data showing impaired Pol III transcription in response to foreign DNA, combined with the recessive mode of inheritance, suggest a different disease mechanism than in patients with VZV susceptibility. Despite immune dysfunction being the main feature, this phenotype could be related to the role of Pol III in nuclear transcription. As with other POLR3-related disorders, further investigations will help delineate the contribution of cytoplasmic and nuclear functions of Pol III to immune phenotypes.

## Conclusion

The molecular-phenotypic relationships that may explain the development of POLR3-related disorders are as of yet only fragmentarily understood. The phenotypic heterogeneity, vast distribution of the mutations known so far across six subunits of Pol III and the resulting mutation-specific structural changes, as well as effects on transcription, argue against a single unifying disease-causing mechanism.

Although formal evidence is still lacking that reduction of Pol III transcript levels is the triggering factor for pathogenesis of POLR3-related disorders, it seems clear that affected neuroanatomical structures and their cellular components have particular vulnerabilities to impaired Pol III transcription or to altered amino acid loading of tRNAs. Mutations in genes encoding the TFIIIB-β component BRF1, Pol III subunits and aminoacyl-tRNA synthetases suggest that perturbations of protein synthesis and proper delivery of protein products to membranes, and possibly ectopic translation in neurons and oligodendrocytes, may play special roles in the development of POLR3-related disorders. Therefore, a dedicated analysis of the transcription of the tRNA, 7SL, and BC200 RNA genes is also necessary to obtain an integral picture of these diseases and their causes, as well as to generate therapeutic strategies for the future. Furthermore, small interspersed nuclear elements (SINEs) and especially the Alu gene subgroup might also play a role in the development of POLR3-HLD, since their promoters and RNA products show sequence and structural similarities to 7SL and BC200 RNAs. However, to the best of our knowledge, their involvement in POLR3-related disorders has not yet been investigated.

Surprisingly, and independently of POLR3-related neurological disorders, mutations in four Pol III subunits affecting innate immune defense have also been described. Apparently, the key factor in the development of these diseases is the activity of the Pol III enzyme in the cytoplasm rather than the transcription of specific Pol III target genes. It will also be of interest for this spectrum of diseases to determine the exact underlying mechanism in order to develop potential therapies to reduce the risk of life-threatening complications from viral infections.

In summary, Pol III transcription has emerged as a key factor in the pathogenesis of several rare debilitating diseases. The establishment of molecular-pathological correlations will facilitate the development of rational therapies in the future.
